# Engineered EVs from LncEEF1G - overexpressing MSCs promote fibrotic liver regeneration by upregulating HGF release from hepatic stellate cells

**DOI:** 10.1038/s12276-025-01413-4

**Published:** 2025-03-03

**Authors:** Jiebin Zhang, Xiaotong Qiu, Yunguo Lei, Haitian Chen, Dongwei Wu, Tingting Wang, Xin Sui, Jiaqi Xiao, Chenhao Jiang, Huayao Zhang, Yasong Liu, Xiaoquan Liu, Yingcai Zhang, Xu Che, Ye Lin, Jia Yao, Zihao Pan, Rong Li, Jun Zheng

**Affiliations:** 1 Department of Hepatic Surgery and Liver Transplantation Center of the Third Affiliated Hospital of Sun Yat-sen University; Organ Transplantation Research Center of Guangdong Province, Guangdong Province Engineering Laboratory for Transplantation Medicine, Guangzhou, China; 2 Guangdong Provincial Key Laboratory of Liver Disease Research, Third Affiliated Hospital of Sun Yat-sen University, Guangzhou, China; 3 Key Laboratory of Integrated Oncology and Intelligent Medicine of Zhejiang Province, Hangzhou First People’s Hospital, Zhejiang University School of Medicine, Hangzhou, China; 4 Surgical ICU, the Third Affiliated Hospital of Sun Yat-sen University, Guangzhou, China; 5 Shaoguan Maternal and Child Health Hospital, Shaoguan, Guangdong China; 6 Department of Infectious Diseases, Third Affiliated Hospital of Sun Yat-sen University, Guangzhou, Guangdong China; 7 Department of Hepatobiliary and Pancreatic Surgery, National Cancer Center/National Clinical Research Center for Cancer/Cancer Hospital and Shenzhen Hospital, Chinese Academy of Medical Sciences and Peking Union Medical College, Shenzhen, China; 8 Department of General Surgery, Guangdong Provincial People’s Hospital, Guangdong Academy of Medical Sciences, Southern Medical University, Guangzhou, China

**Keywords:** Mesenchymal stem cells, Transcription, Nanobiotechnology

## Abstract

Fibrosis is a disease that negatively affects liver regeneration, resulting in severe complications after liver surgery. However, there is still no clinically effective treatment for promoting fibrotic liver regeneration because the underlying hepatocellular mechanism remains poorly understood. Through microRNA microarrays combined with the application of AAV6, we found that high expression of miR-181a-5p in activated hepatic stellate cells (HSCs) suppressed the expression of hepatic growth factor (HGF) and partially contributed to impaired regeneration potential in mice with hepatic fibrosis that had undergone two-thirds partial hepatectomy. As nanotherapeutics, mesenchymal stem-cell-derived extracellular vesicles (MSC-EVs) have been verified as effective treatments for liver regeneration. Here we observe that MSC-EVs can also promote fibrotic liver regeneration via enriched lncEEF1G, which acts as a competing endogenous RNA to directly sponge miR-181a-5p, leading to the upregulated expression of HGF in HSCs. Finally, engineered MSC-EVs with high expression of lncEEF1G (lncEEF1G^OE^-EVs) were constructed, suggesting greater potential for this model. In summary, our findings indicate that lncEEF1G^OE^-EVs have a nanotherapeutic effect on promoting regeneration of fibrotic livers by modulating the miR-181a-5p/HGF pathway in HSCs, which highlights the potential of extracellular vesicle engineering technology for patients with hepatic fibrosis who have undergone hepatic surgery.

**Figure:**
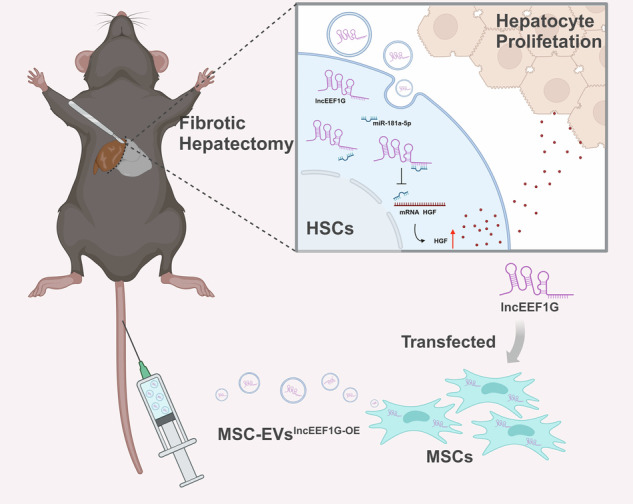
Engineered mesenchymal stem cells that overexpress lncEEF1G can secrete extracellular vesicles that are rich in lncEEF1G (lncEEF1G^OE^-EVs). Upon injection of lncEEF1G^OE^-EVs into a fibrotic 70% partial hepatectomy mouse model, lncEEF1G competitively binds to miR-181a-5p in hepatic stellate cells, preventing the interaction between miR-181a-5p and the messenger RNA of hepatocyte growth factor. This consequently leads to an increase in the secretion of hepatocyte growth factor and the promotion of hepatocyte proliferation.

Engineered mesenchymal stem cells that overexpress lncEEF1G can secrete extracellular vesicles that are rich in lncEEF1G (lncEEF1G^OE^-EVs). Upon injection of lncEEF1G^OE^-EVs into a fibrotic 70% partial hepatectomy mouse model, lncEEF1G competitively binds to miR-181a-5p in hepatic stellate cells, preventing the interaction between miR-181a-5p and the messenger RNA of hepatocyte growth factor. This consequently leads to an increase in the secretion of hepatocyte growth factor and the promotion of hepatocyte proliferation.

## Engineered extracellular vesicles enhance liver regeneration

Partial hepatectomy is a common treatment for liver diseases, but liver fibrosis can hinder recovery. This study explores how mesenchymal stem-cell-derived extracellular vesicles (MSC-EVs) might help fibrotic livers regenerate after partial hepatectomy. Researchers found that MSC-EVs can boost liver regeneration by increasing hepatocyte growth factor production in hepatic stellate cells. The study uses a mouse model with liver fibrosis induced by carbon tetrachloride and then performed a partial hepatectomy. Researchers isolated MSCs from umbilical cords and extracted EVs from these cells. They injected these MSC-EVs into the mice and observed their effects on liver regeneration. MSC-EVs were found to be taken up by hepatic stellate cells, leading to increased hepatocyte growth factor production, which is crucial for liver cell proliferation. The results suggest MSC-EVs could be a promising treatment to enhance liver regeneration in fibrotic conditions.

This summary was initially drafted using artificial intelligence, then revised and fact-checked by the author.

## Introduction

Partial hepatectomy (PH) is an effective treatment for multiple liver diseases because of the excellent regenerative capacity of the liver, which causes hepatocytes to rapidly proliferate to avoid tissue loss after PH. Therefore, the proliferative potential of hepatocytes is an essential determinant of the therapeutic effect and postoperative prognosis of PH^1^. Several risk factors negatively affect hepatocellular proliferation, which can increase patients’ susceptibility to small for size syndrome and result in a significantly increased rate of mortality after PH. Overall, liver fibrosis, which can be caused by multiple etiologies (including virus infection, alcohol and drugs), is one of the primary causes of the rapid decline in the regenerative capacity of the liver^2,3^. However, the underlying mechanism by which liver fibrosis limits hepatocellular proliferation remains poorly defined, resulting in few therapeutic approaches available to clinicians for the promotion of fibrotic liver regeneration after PH.

Liver fibrosis is regulated by dynamic interactions between nonparenchymal and parenchymal cells, among which hepatic stellate cells (HSCs), the major subcluster of liver-resident fibrogenic cells, play a prominent role in the scaring process^4^. Previously, we demonstrated the central role of HSCs activation in liver fibrosis, which is characterized by fibrogenic myofibroblastic features, including proliferation, contraction and production of the extracellular matrix (ECM)^5^. Interestingly, the role of HSCs in liver regeneration is bidirectional: HSCs release hepatocyte growth factor (HGF) to support a regeneration response, whereas transforming growth factor beta (TGF-β) delays this response^2^. Therefore, the balance of HGF and TGF-β secreted by HSCs may be a therapeutic target for promoting fibrotic liver regeneration after PH.

As they possess specific regeneration, immunomodulation and multiple differentiation capabilities, mesenchymal stem cells (MSCs) have gradually gained attention as promising cell therapies for various diseases. Previously, our experimental studies demonstrated the potential of MSCs not only in alleviating hepatic ischemia/reperfusion injury (HIRI) via immunomodulation and increased mitophagy to restore the mitochondrial function of hepatocytes but also in suppressing inflammasome activation in macrophages to attenuate acute lung injury^6–9^. In a clinical study, we reported that MSCs could aid in treating severe postoperative complications after liver transplantation and improving patient prognosis after ABO-incompatible liver transplantation^7^. Moreover, extracellular vesicles (EVs), which are 30–300 nm nanosized vesicles that carry numerous functional components, have increasingly been identified as the dominant way by which MSCs perform their biological functions. As a type of nanotherapy, MSC-derived EVs (MSC-EVs) play biological roles similar to those of parental MSCs and additionally exhibit several unique advantages, including high biosafety and low rates of pulmonary embolism and tumorigenicity. Our previous data revealed that MSC-EVs have the potential to limit oxidative stress and can attenuate HIRI via immunoregulation^10–12^. Recently, we revealed that MSC-EVs promote aged liver regeneration by promoting hepatocellular mitophagy^13^. Other studies have also demonstrated the roles of MSC-EVs in protecting against liver fibrosis by regulating the activity and function of HSCs^14,15^. However, the therapeutic effects and corresponding underlying mechanisms of MSC-EVs in fibrotic liver regeneration remain largely unknown.

The current study aims to explore the potential of MSC-EVs for the treatment of fibrotic livers subjected to PH, and we found that MSC-EVs promote fibrotic liver regeneration by increasing the synthesis and release of HGF in HSCs. Mechanistically, lncEEF1G enriched in MSC-EVs could be transferred into HSCs and subsequently act as a miR-181a-5p sponge to upregulate HGF expression. Notably, lncEEF1G-rich EVs (lncEEF1G^OE^-EVs) isolated from genetically modified MSCs were utilized to further stimulate HGF secretion by HSCs, highlighting a promising treatment involving EV-mediated specific long noncoding RNA (lncRNA) delivery to promote fibrotic liver regeneration.

## Methods and materials

### Animals

The 8–10-week-old C57BL/6 mice (male, 22–25 g in weight) used in this study were obtained from the Model Animal Research Center of Nanjing University. The mice were raised in a specific pathogen-free environment with 50% humidity and 22 °C with a 12–12 h light–dark cycle at the Experimental Animal Center of Sun Yat-sen University and were provided standard laboratory food and water abiding by the Guideline of Sun Yat-sen University for Animal Experimentation. The animal studies were approved by the Animal Care and Use Committee of the Third Affiliated Hospital of Sun Yat-sen University and were performed in compliance with the National Institutes of Health Guide for the Care and Use of Laboratory Animals.

### Preparation of a 70% fibrotic PHx model

To prepare a mouse fibrotic PH (PHx) model, the mice were first treated with 20% carbon tetrachloride (CCl_4_) (5 μl g^−1^ (CCl_4_:mineral oil ratio of 1:4)) two times per week for 2 weeks, after which the PHx model was prepared according to the standardized procedure described in a previous study^15^. In brief, after anesthetization via inhalation of 2% isoflurane, a midline laparotomy incision was made, and 70% of the liver, covering the median and left lateral lobes, was subsequently removed by tying the related hepatic artery, bile duct and portal vein with 4–0 silk. After nonactive bleeding was detected, the abdominal cavity was closed.

### Measurement of serum hepatic enzymes

The concentrations of serum hepatic enzymes, including alanine aminotransferase (ALT), aspartate aminotransferase (AST) and lactate dehydrogenase (LDH), were measured via a 7180 Biochemical Analyzer (Hitachi).

### Ratio of LW/BW

The degree of liver regeneration was evaluated by calculating the liver weight (LW)-to-body-weight (BW) ratio (LW/BW) at each time point.

### H&E, immunohistochemistry and PSR staining

The liver tissues were collected, fixed with neutral-buffered formalin (10%), embedded in paraffin for 24 h and then cut into 4-μm-thick sections. For histologic examination, hematoxylin and eosin (H&E) staining was performed under a light microscope (Nikon, E100) by an observer who was blinded to the experimental group design. To detect the degree of fibrosis and count the Ki67-positive cells in the liver, after deparaffinization and rehydration, the sections were treated with sodium citrate buffers (pH 6.0, 10 mM) for antigen retrieval, blocked with bovine serum albumin (5%) and incubated with primary antibodies (α-SMA or Ki67; Abcam) at 4 °C overnight. A secondary antibody was subsequently used to treat the sections for 30 min at room temperature, followed by nuclear staining with 3,3′-diaminobenzidine (Agilent). For picrosirius red (PSR) staining, the sections were incubated with PSR solution at room temperature for 1 h and then with hematoxylin for 5 min.

### Isolation of primary HSCs

The standard procedures for isolating primary HSCs from the mice were performed as previously described^16^. In brief, collagenase type IV (Gibco, Life Technologies) with DNase (Sigma) and pronase (Roche) were used for enzymatic digestion, followed by centrifugation in density gradient medium to purify the primary HSCs (Supplementary Fig. 1a). The cells were cultured in Dulbecco’s modified Eagle medium (DMEM) containing penicillin–streptomycin (1%), fetal bovine serum (FBS, 10%, PAN-Biotech) and glutamine (1%) at 37 °C with 5% CO_2_. Retinoid fluorescence was used to identify primary HSCs (Supplementary Fig. 1b).

### Western blotting

The samples were lysed using cold radioimmunoprecipitation buffer supplemented with sodium deoxycholate (10%), Triton X-100 (0.1%), Tris–HCl (50 mM, pH 7.4), sodium dodecyl sulfate (10%), ethylene diamine tetraacetic acid (2 mM), NaCl (150 mM) and protease cocktail inhibitor (KeyGEN BioTECH). Next, the the concentrations of the proteins were detected, and the same amounts of proteins were subjected to 12% sodium dodecyl sulfate–polyacrylamide gel electrophoresis to separate the proteins, which was followed by transfer to polyvinylidene difluoride membranes (Millipore). After being blocked with nonfat milk (5%) at room temperature for 1 h, the membranes were incubated with primary antibodies (including antibodies against Proliferating Cell Nuclear Antigen (PCNA) (1:1,000; Cell Signaling Technology), HGF (1:1,000; Cell Signaling Technology), TGF-β (1:1,000; Cell Signaling Technology), apoptosis-linked gene-2 interacting protein X (ALIX) (1:1,000; Cell Signaling Technology), CD63 (1:1,000; Abcam), CD81 (1:1,000; Cell Signaling Technology), glucose-regulated protein 94 (GRP94) (1:1,000; Cell Signaling Technology), α-SMA (1:1,000; Abcam), Vimentin (1:1,000; Abcam) and β-actin (1:1,000; Cell Signaling Technology)) overnight at 4 °C, after which they were incubated with secondary antibodies (anti-rabbit IgG, Sigma-Aldrich; anti-mouse IgG, Sigma-Aldrich) for 1 h at room temperature on a table shaker. The blots were treated with an enhanced chemiluminescence substrate (ECL, Merck KGaA) and visualized with a ChemiDoc MP Imaging System (Bio-Rad).

### Total RNA extraction and RT‒qPCR

The total RNA of each sample was extracted using TRIzol (Invitrogen, Life Technology) following the manufacturer’s instructions. After the purity and concentration were measured via a ultraviolet‒visible spectrophotometer (BIOMATE 3S, Thermo Scientific), a Transcriptor First Strand complementary DNA Synthesis Kit (Roche Applied Science) was used to reverse transcribe the RNA to cDNA, followed by amplification of the cDNA templates via a PCR Thermal Cycler (Bio-Rad). Quantitative real-time polymerase chain reaction (RT‒qPCR) was conducted using SYBR Master Mix (Roche Applied Science) and detected via a reverse transcription system (LC-480, Roche). The sequences of the primers used were designed and constructed by RiboBio (Guangzhou, China), and β-actin was used as a housekeeping gene. The sequences of all the primers used in this study are listed in Supplementary Table 1.

### Enzyme-linked immunosorbent assay

The concentration of HGF in the supernatants derived from the in vitro experiments was measured via an enzyme-linked immunosorbent assay (ELISA) kit according to the manufacturer’s protocol (R&D Systems).

### Cell culture and induction of HSC activation in vitro

The immortalized human HSC cell line LX-2, which was obtained from the Cell Bank of the China Academy of Sciences, was cultured in DMEM (high glucose, 4.5 g l^−1^) supplemented with 10% FBS and maintained in a 37 °C incubator with 5% CO_2_. To activate HSCs, LX-2 cells were cultured in serum-free DMEM for 12 h, followed by treatment with TGF-β for 6 h.

### Microarray analysis of miRNAs

The total RNA of the HSCs in each group was extracted using TRIzol according to the manufacturer’s protocol. After the quantity and quality of the RNA were measured via a NanoDrop ND-1000 and the integrity of the RNA was evaluated via standard denaturing agarose gel electrophoresis, sample labeling and array hybridization were performed using the Agilent microRNA (miRNA) Microarray System and following the miRNA Complete Labeling and Hyb Kit protocol (Agilent Technology). In brief, Cyanine 3-pCp was used to label the total miRNA of each sample under the action of T4 RNA ligase. The labeled cRNA was concentrated, desiccated and redissolved with water, fragmented with a blocking agent and fragmentation buffer, heated for 30 min at 60 °C and finally diluted with GE hybridization buffer. One hundred microliters of hybridization solution was dispensed into the gasket slide, which was subsequently placed on the gene expression microarray slide for 17 h of incubation at 65 °C in an Agilent hybridization oven. After being washed, the hybridized arrays were fixed and scanned with an Agilent Microarray Scanner.

The acquired array images were analyzed via Agilent Feature Extraction software (version 11.0.1.1). The GeneSpring GX v12.1 software package (Agilent Technology) was used to perform quantile normalization and data processing. After that, the miRNAs whose flags were detected (‘All Targets Value’) were selected for further analysis. Differential miRNAs with statistical significance between the HSCs, and activated HSCs were identified through volcano plot filtering. Hierarchical clustering was conducted via R scripts.

### Dual-luciferase reporter assay

To investigate the combination of miR-181a-5p with the targeted gene HGF, the 3′ untranslated region (UTR) of HGF with a wild-type (WT) binding site or corresponding mutant site was inserted into a luciferase reporter gene vector (GeneChem). The miR-181a-5p mimic or NC combined with the constructed HGF plasmids (HGF-3′UTR-WT or HGF-3′UTR-Mut) were cotransfected into HEK-293T cells via Lipofectamine 3000 (Invitrogen) according to the manufacturer’s protocol. After 48 h of transfection, the Renilla and firefly luciferase activities were analyzed via a dual-luciferase reporter assay system (Promega) according to the manufacturer’s protocol. Renilla luciferase was used as an internal control. The results are presented as a ratio.

In addition, to further determine the binding between lncEEF1G and miR-181a-5p, the sequences of lncEEF1G with the WT miR-181a-5p binding site or corresponding mutant site were also inserted into the luciferase reporter gene vector (WT hsa_lncEEF1G and mutant hsa_lncEEF1G). Then, the related plasmids and miR-181a-5p mimics were cotransfected into HEK-293T cells. The firefly and Renilla luciferase activities were also used to evaluate the interaction between lncEEF1G and miR-181a-5p.

### miRNA treatment

miR-181a-5p mimics with nontarget control small interfering RNA (NC-mimics) and a miR-181a-5p inhibitor with a negative control inhibitor (NC-inhibitor) were synthesized by RiboBio. When the cells reached 80–90% confluency, they were transfected with miR-181a-5p mimics for overexpressing miR-181a-5p or with a miR-181a-5p inhibitor for knockdown via Lipofectamine 2000 (Invitrogen) according to the manufacturer’s instructions.

### AAV6 therapy

A triple-transfection, helper-free approach was used to package recombinant adeno-associated virus-6 (AAV6) with the double-stranded CMV bGlobin-eGFP-U6-mmu-miR-181a-5p TuD (AAV6-miR-181a-5p inhibitor) to specifically inactivate miR-181a-5p in HSCs in vivo, which was purified by Obio Technology. qPCR was performed to determine the titer of the recombinant AAV. AAV6-eGFP-mmu-miR-181a-5p TuD or AAV6-eGFP-shControl (200 μl of solution containing 1.5 × 10^11^ particles of AAV vectors; Hanbio Biotechnology) was injected through the caudal vein. The distribution of AAV6 was monitored via a Bruker small animal optical imaging system. HSCs were subsequently isolated for flow cytometry analysis to detect the degree of AAV6 uptake by the HSCs, and qPCR was used to measure the efficiency of miR-181a-5p inactivation.

### Isolation, culture and identification of human umbilical cord-derived MSCs

The isolation and culture of MSCs were performed under sterile conditions according to standardized protocols, which were approved by the Research Ethics Committee of the Third Affiliated Hospital of Sun Yat-sen University, with approval granted on 30 December 2020 (approval number 2020-14)^9^. All enrolled participants provided informed consent. After the remaining blood in the umbilical cords was removed with cold phosphate-buffered saline (PBS), the umbilical cords were cut into 10 mm^3^ pieces and subsequently placed in type I collagenase containing 3 mM CaCl_2_ and 0.1% hyaluronidase for 4 h of digestion at 37 °C, followed by replacement with low-glucose DMEM supplemented with FBS (10%, PAN-Biotech) and maintenance in humidified, 5% CO_2_ conditions at 37 °C. To remove nonadherent cells, the medium was changed every 3 days.

To detect MSC-related surface characteristics, the cells were collected, washed with PBS containing bovine serum albumin (1% BSA; Sigma-Aldrich) to block nonspecific antigens and incubated with monoclonal antibodies (FITC‒CD11b, PE‒CD105, FITC‒CD90, FITC‒HLA‒DR, PE‒CD73, FITC‒CD34, FITC‒CD19 and FITC‒CD45) for 30 min at 4 °C in the dark. A 13-color FACS Calibur (Beckman Coulter) was used to measure the fluorescence intensity, which was analyzed with FlowJo software (TreeSta).

Specific medium for adipogenesis and osteogenesis (Gibco, Life Technologies) was used to detect the ability of the MSCs to undergo adipogenic and osteogenic differentiation. After 21 days of culture, the cells were stained with Oil Red O or Alizarin Red S to assess their adipogenesis and osteogenesis abilities.

### Purification, identification and quantification of MSC-EVs

The medium used to culture the MSCs was changed to low-glucose DMEM supplemented with exosome-free FBS (10%), and the cells were incubated for 48 h after they reached 70–80% confluency. The supernatant was collected for centrifugation at 300*g* and 3,000*g* for 10 min at 4 °C to discard the residual cells and dead cells, and then the supernatant was centrifuged at 10,000*g* and 4 °C for 30 min to remove cellular debris. Two ultracentrifugation steps at 100,000*g* for 70 min at 4 °C were subsequently performed to purify the MSC-EVs. The total protein content of the MSC-EVs was measured via a bicinchoninic protein assay kit (KeyGEN BioTECH) following the manufacturer’s protocol.

Transmission electron microscopy (TEM) was performed to visualize the MSC-EVs according to previous studies^10^. In brief, the MSC-EVs were fixed in 2.5% glutaraldehyde for 1 h at 4 °C, ultracentrifuged, resuspended in PBS and dripped on a formvar/carbon-coated grid. After 3% aqueous phosphor-tungstic acid was used for negative staining, the samples were investigated via TEM.

A nanoparticle tracking analysis (NTA) was conducted to analyze the size distribution of the MSC-EVs by a Zetaview PMX 120 (Particle Metrix) with an sCMOS camera. The MSC-EVs were diluted with PBS and detected under an optical microscope perpendicular to the beam axis. NTA software (Zetaview, version 8.05.14 SP7) was used to analyze the results.

### Uptake of MSC-EVs by HSCs

PKH26 Cell Membrane Labeling Dye (Sigma-Aldrich) was used to label MSC-EVs following the manufacturer’s protocol. After that, PKH26-labeled MSC-EVs were incubated with HSCs for 2 h to investigate the role of MSC-EVs uptake by HSCs. The cells were washed twice with PBS, fixed in 4% paraformaldehyde for 30 min at 4 °C, stained with DAPI for 2 min in the dark and visualized under a Zeiss 880 confocal microscope (Nikon Instruments).

### Tracking MSC-EVs in vivo

To detect the distribution of the MSC-EVs in the fibrotic PHx model, the MSC-EVs were labeled with 1,1-dioctadecy-3,3,3-tetramethy-lindotricarbocyanine iodide lipophilic dye (DiR, Thermo Fisher Scientific) for 15 min in the dark at room temperature, followed by ultracentrifugation according to the MSC-EVs isolation methods to obtain DiR-labeled MSC-EVs. After the PHx model was prepared, DiR-labeled MSC-EVs were immediately transferred through the caudal vein. The biodistribution of the MSC-EVs was directly observed by placing the mice in a Bruker small animal optical imaging system (In Vivo Xtreme II) at the selected time points after EVs administration. In addition, five organs, including the heart, lung, liver, spleen and kidneys, of the mice in each group were collected to assess the fluorescence intensity of the labeled EVs via the same system.

### MSC-EV treatment

LncEEF1G^OE^-EVs were obtained from MSCs transfected with lncEEF1G shRNA lentivirus, and MSC-EVs were obtained by the method described above. After the 70% PHx model was prepared, a single injection of MSC-EVs (100 µl, approximately 1 × 10^8^ nanoparticles), lncEEF1G^OE^-EVs (100 µl, approximately 1 × 10^8^ nanoparticles) or PBS (100 µl) was administered via the tail vein. The postoperative BW of each mouse was recorded daily, and the mice were killed, after which the liver tissues and serum were collected at specified time points.

### LncRNA profiling analysis of MSC-EVs

For RNA isolation as well as library preparation and sequencing, three independent replicates of MSC-EVs were collected, an exoRNeasy Maxi Kit (Qiagen) was used to purify the total exosome-derived RNA of each sample, and an RNA Nano 6000 Assay Kit for the High Agilent Bioanalyzer 2100 system (Agilent Technologies) was used to examine RNA integrity. After the genomic DNA was removed from the total RNA, the first-strand cDNA was reverse-transcribed to the second strand and then subjected to end repair, A-tailing and adapter ligation and purification. The ribosomal RNA was removed from the first round of amplification via gene-specific lncDA-C primers, after which the library fragments were purified via the AMPure XP system to select cDNA fragments 250–300 bp in length. The second round of PCR amplification was conducted to purify the library. The concentration and the insert size of the obtained library were determined by a Qubit fluorometer and an Agilent 2100 Bioanalyzer, respectively, which was followed by measuring the concentration of the cDNA library again via PCR. Once the insert size and concentration of the acquired library were confirmed, the samples were subjected to Illumina sequencing.

For the data analysis, the raw reads were first processed to obtain the clean reads through in-house Perl scripts to deplete the following reads: (1) without an insert sequence or 3′ adapter, (2) with more than 10% N,)3) with a 5′ adapter, (4) with poly A/T/G/C and (5) with more than 50% nucleotides with Qphred ≤20. The adapter sequences from the 3′ ends of the reads were removed, and the Q20, Q30 and GC contents were subsequently calculated. The clean reads were mapped to a reference genome via HISAT2 software, and the read alignment results were assembled and transferred to the program StringTie. After the transcripts were merged via Cuffmerge software, all the lncRNAs were identified by removing (1) the transcripts of low expression with an FPKM <0.5, (2) short transcripts <2 exons and <200 bp, (3) the transcripts with protein-coding abilities via the CNCI, Pfam and CPC2 databases and (4) the transcripts mapped within the flanking regions (1 kb) of an annotated gene via Cuffcompare. The novel lncRNAs were named according to the rules of the HUGO Gene Nomenclature Committee (HGNC) and were compared with known lncRNAs and messenger RNAs. StringTie software was used to quantify the transcripts and genes, after which reads per kilobase of transcript per million mapped reads were obtained. Cuffdiff was used to perform differential expression analysis, and the *P* values were modulated via Benjamini and Hochberg’s approach to control the false discovery rate. When adjusted *P* < 0.05 and |log_2_ (fold change)| >0, the genes were considered differentially expressed. Finally, target gene prediction of the lncRNAs was performed via *cis*-acting target gene prediction and *trans*-acting target gene prediction.

### RIP

The cells were crosslinked with 1% paraformaldehyde in ice-cold PBS for 10 min, lysed in radioimmunoprecipitation lysis buffer and incubated with Dynabeads protein G combined with anti-IgG or anti-AGO2 antibodies followed by rotation at 4 °C overnight. Then, TRIzol reagent was used to purify the immunoprecipitated RNAs, which were detected via RT‒qPCR with specific primers.

### Biotin-labeled miRNA capture

The RNA pulldown was conducted according to a previous study^17^. Briefly, MSCs stably overexpressing lncEEF1G were transfected with the biotin-labeled miR-181a-5p mimic (GenePharma) for 48 h. After being blocked with yeast tRNA at 4 °C for 2 h, the cell lysates were incubated with streptavidin-Dyna beads M-280 at 4 °C overnight to pull down the biotin-coupled RNA complex. The abundance of lncEEF1G was measured via RT‒qPCR.

### RNA FISH

RNA fluorescence in situ hybridization (FISH) was performed to observe the colocalization of lncEEF1G (RiboBio) and miR-181a-5p (BersinBio) in cells via a Fluorescent in Situ Hybridization Kit (RiboBio) following the manufacturer’s protocols. The cells were visualized under a Zeiss 880 confocal microscope (Nikon Instruments).

### LncEEF1G shRNA lentivirus transfection

ShlncEEF1G (shlncEEF1G-1, shlncEEF1G-2 and shlncEEF1G-3) and its negative control were designed and synthesized by General Biosystems. The constructed plasmids and corresponding packaging plasmids were subsequently transfected into HEK-293T cells via Lipofectamine 2000 (Invitrogen) following the manufacturer’s instructions. After 4 days of transduction, the number of GFP-positive cells was measured via fluorescence microscopy (Zeiss).

The serum-free medium with shlncEEF1G or null shRNA was added to the MSCs for 48 h, followed by replacement with fresh culture medium for another 48 h of incubation. To screen the optimal shRNA, the fluorescence intensity was detected and the gene transfection efficiency was confirmed via RT‒qPCR.

### Statistical analysis

The data in the current study are shown as direct values or means ± standard deviations (s.d.). GraphPad Prism 11 software (GraphPad Software) was used to perform the statistical analysis. As appropriate, group comparisons were performed via one-way analysis of varianceor an unpaired two-tailed Student’s *t*-test. The in vitro experiments were independently repeated three times. A probability level of a *P* value less than 0.05 (*P* < 0.05) was recognized as statistically significant.

## Results

### Liver regeneration is weakened in fibrotic mice

**Fig. 1:**
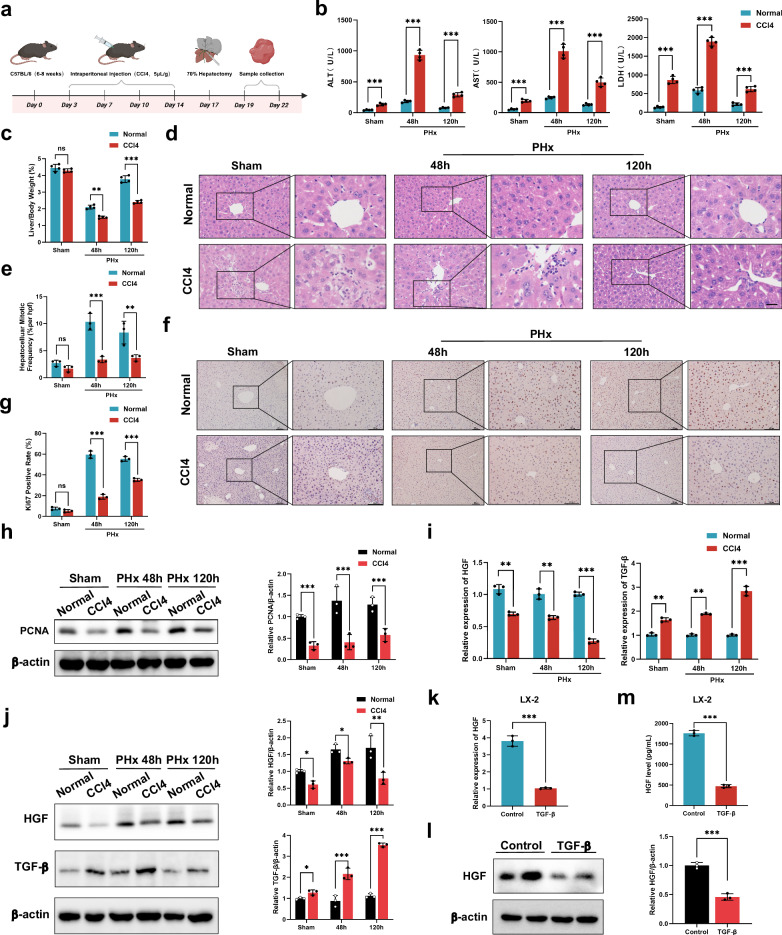
Liver regeneration is weakened in fibrotic mice. **a** A schematic diagram of the preparation of a mouse hepatic fibrotic model through CCl_4_ treatment followed by two-thirds PHx. **b** Serum ALT, AST and LDH levels at the indicated time points after PHx (*n* = 4 independent biological mouse samples). **c** LW/BW ratios at the indicated time points after PHx (*n* = 4 independent biological mouse samples). **d** The representative images of H&E-stained samples at the indicated time points after PHx. Scale bar, 50 μm. **e** A quantification of the mitotic frequency determined by counting the numbers of mitotic nuclei and total nuclei in randomly selected fields (*n* = 3 independent experiments). **f**, **g** The representative images (**f**) and quantification (**g**) of Ki67 immunohistochemical staining at the indicated time points after PHx. Scale bar, 100 μm (*n* = 3 independent experiments). **h**, Western blotting (left) and relative quantification (right) of PCNA protein expression at the indicated time points after PHx in liver tissues (*n* = 3 independent biological mouse samples). **i** RT‒qPCR analysis of the levels of HGF and TGF-β in HSCs isolated from the PHx model (*n* = 4 independent cell experiments). **j** Western blotting (left) and relative quantification (right) showing the expression of HGF and TGF-β in HSCs isolated from the PHx model (*n* = 3 independent experiments). **k**–**m** In vitro experiments include: RT‒qPCR analysis of HGF in the LX-2 HSC line (*n* = 3 independent cell experiments) (**k**) western blotting (left) and relative quantification (right) showing the expression of HGF in the LX-2 HSC line (*n* = 3 independent cell experiments) (**l**) and ELISA results showing the HGF levels in LX-2 HSC line culture medium (*n* = 3 independent cell experiments) (**m**). The statistical data are presented as mean ± s.d., and the error bars represent the means of three independent experiments or four independent biological mouse samples. Statistical significance was determined by a Student’s *t*-test. *P < 0.05, **P < 0.01 and ***P < 0.001. n.s., no significance.

To investigate the regenerative potential of the fibrotic liver, we first prepared a mouse hepatic fibrotic model via CCl_4_ treatment and then performed a two-thirds PHx (Fig. 1a). As shown in Supplementary Fig. 2a, the livers presented rough and hard surfaces after two weeks of CCl_4_ administration. The normal hepatic lobule structure was disrupted by the irregular arrangement of hepatocytes, which were substituted with pseudolobules (Supplementary Fig. 2b). PSR staining revealed increased levels of fibrin in the fibrotic liver (Supplementary Fig. 2c). As α-SMA and Vimentin are markers of HSCs activation, we also detected high expression of α-SMA and Vimentin in these liver tissues (Supplementary Fig. 2d). We subsequently used this model to further prepare a fibrotic PHx model, and liver and serum samples were collected after 48 and 120 h. Compared with those in nonfibrotic mice, the levels of hepatic enzymes, including ALT, AST and LDH, were significantly greater in fibrotic mice that underwent two-thirds hepatectomy (Fig. 1b). An increasing LW/BW ratio was detected in the nonfibrotic group from 48 to 120 h, whereas it was lower in the fibrotic groups (Fig. 1c). Liver regeneration was also evaluated by the number of mitotic hepatocytes, Ki67-positive rates and PCNA expression levels, three critical indicators reflecting mitotic activity and cellular proliferation. As shown in Fig. 1d–h, fibrosis substantially suppressed hepatocyte proliferation, as evidenced by the reduced number of mitotic hepatocytes, decreased Ki67 staining and suppressed expression of PCNA compared with those in nonfibrotic mice.

Growing evidence has demonstrated that HSCs significantly affect liver regeneration during fibrosis via secretion of factors such as HGF and TGF-β^2^. Therefore, we further isolated HSCs from the fibrotic PHx model and measured the expression of HGF and TGF-β. Compared with those in the nonfibrotic groups, the level of HGF in the fibrotic groups was lower, and the level of TGF-β in the fibrotic groups was greater (Fig. 1i, j). For the in vitro assays, we used the LX-2 HSC line and activated it with TGF-β according to previous methods^18,19^. Consistently, the activated HSCs exhibited reduced expression of HGF compared with that in the normal group without any treatment (Fig. 1k–m). Taken together, these data show that a lower level of HGF secretion by activated HSCs may partially contribute to weakened liver regeneration in fibrotic mice.

### The upregulation of miR-181a-5p in activated HSCs blocks HGF expression

**Fig. 2:**
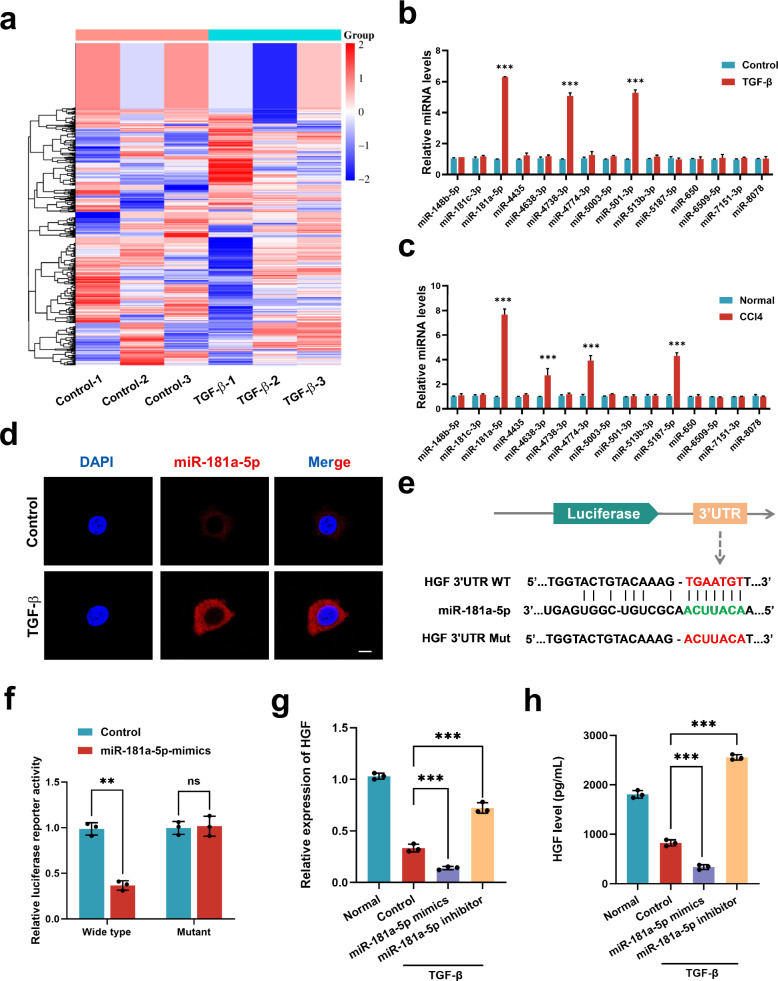
The upregulation of miR-181a-5p in activated HSCs blocks HGF expression. **a** A heat map displaying the expression patterns of miRNAs in control and TGF-β-treated HSCs. The upregulated genes are shown in red, and the downregulated genes are shown in blue (*n* = 3 independent cell samples). **b** RT‒qPCR results showing the levels of differential miRNA expression in the HSC lines of each group (*n* = 3 independent cell samples). **c** RT‒qPCR results showing the levels of differential miRNA expression in the liver tissue of each group (*n* = 3 independent biological mice samples). **d** FISH results showing the localization of miR-181a-5p (red) in control and TGF-β-treated HSCs. Scale bar, 20 μm. **e** A schematic diagram of the construction of luciferase reporter plasmids with mutant and WT HGF sequences. **f** The luciferase activity of WT and mutant HGFs was detected by luciferase reporter assays after cotransfection with either the miR-181a-5p or control mimics (*n* = 3 independent experiments). **g**, **h** RT‒qPCR (**g**) and ELISA (**h**) results showing the levels of HGF in each HSC treatment group (*n* = 3 independent cell experiments). The statistical data are presented as mean ± s.d., and the error bars represent the means of three independent experiments. Statistical significance was determined by a Student’s *t*-test. *P < 0.05, **P < 0.01 and ***P < 0.001. n.s., no significance.

To explore the mechanism underlying low HGF expression by activated HSCs, a wide miRNA microarray profile analysis was carried out. Unsupervised clustering analysis separately grouped HSCs and HSCs treated with TGF-β (activated HSCs), revealing differences in miRNA expression between these two groups (*P* < 0.05) (Fig. 2a). Through an in-depth analysis, 15 miRNAs (miR-148b-5p, miR-181c-3p, miR-4435, miR-4638-3P, miR-4738-3p, miR-4774-3p, miR-5003-5p, miR-501-3p, miR-513b-3p, miR-5187-5p, miR-650, miR-6509-5p, miR-7151-3p, miR-8078 and miR-181a-5p) in total were upregulated, whereas ten miRNAs (miR-1228-5p, miR-1249-5p, miR-2392, miR-335-3p, miR-431-3p, miR-4675, miR-4722-3p, miR-6756-5p, miR-6776-3p and miR-6886-5p) were downregulated in the activated HSCs compared with those in the normal group. In addition, we performed RT‒qPCR assays for both in vitro experiments and in vivo models to further validate the differential miRNA expression, which revealed that the expression of miR-181a-5p was significantly greater in the fibrotic group (activated HSCs group) than in the normal group (Fig. 2b, c). The results of the FISH assays also confirmed that miR-181a-5p was highly expressed in activated HSCs and was located mainly in the cytoplasm (Fig. 2d). On the basis of the above results, we selected miR-181a-5p for further investigation. MiRWalk, an online database, was used to predict the target genes of miR-181a-5p, which suggested that miR-181a-5p may interact with HGF. Then, we constructed luciferase reporter plasmids containing the mutant HGF-3′UTR sequence or the WT HGF-3′UTR sequence (Fig. 2e) and cotransfected them with miR-181a-5p to confirm whether HGF was a direct target gene of miR-181a-5p. The results revealed that the miR-181a-5p mimic led to a greater than 50% reduction in luciferase activity but had no effect on luciferase activity when the cells were transfected with a vector containing the mutant HGF-3′UTR sequence (Fig. 2f). Finally, the RT‒qPCR and ELISA results revealed that the expression and secretion of HGF by HSCs were downregulated or upregulated after transfection with the miR-181a-5p mimic or inhibitor, respectively (Fig. 2g, h and Supplementary Fig. 3a). Taken together, these findings indicate that miR-181a-5p is highly expressed in activated HSCs and can bind to HGF to downregulate its expression.

### Blocking miR-181a-5p in HSCs strengthens liver regeneration in the fibrotic PHx model

**Fig. 3:**
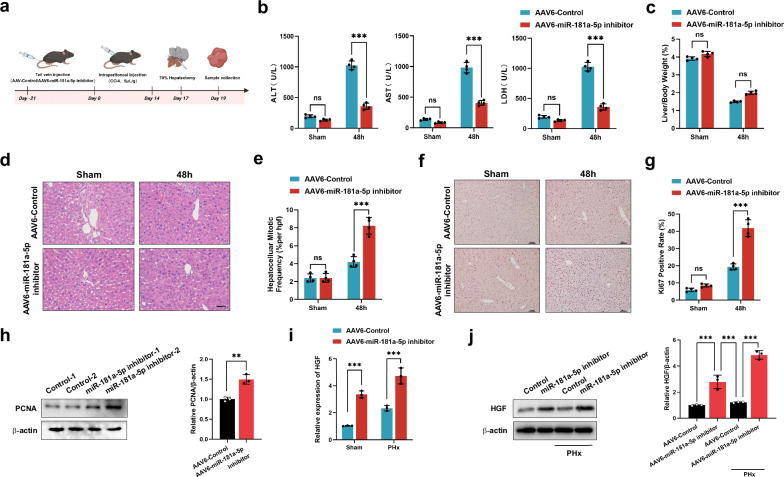
Blocking miR-181a-5p in HSCs strengthens liver regeneration in fibrotic PHx model mice. **a** A schematic diagram of the application of an AAV6-miR-181a-5p inhibitor to determine whether miR-181a-5p can modulate the expression of HGF in HSCs and affect the regeneration of the fibrotic liver. **b** Serum ALT, AST and LDH levels in different treatment groups (*n* = 4 independent biological mouse samples). **c** The ratio of LW/BW in different treatment groups (*n* = 4 independent biological mouse samples). **d** The representative images of H&E-stained samples from different treatment groups. Scale bar, 50 μm. **e** A quantification of the mitotic frequency determined by counting the numbers of mitotic nuclei and total nuclei in randomly selected fields (*n* = 4 independent biological mouse samples). **f**, **g** The representative images (**f**) and quantification (**g**) of Ki67 immunohistochemical staining in different treatment groups. Scale bar, 100 μm (*n* = 4 independent biological mouse samples). **h** Western blotting (left) and relative quantification (right) showing the expression of PCNA in different treatment groups (*n* = 3 independent experiments). **i**, **j** RT‒qPCR (**i**) and representative western blotting (**j**, left) results showing the expression of HGF in HSCs isolated from different treatment groups and a quantification of HGF protein levels (**j**, right) (*n* = 3 independent experiments). The statistical data are presented as mean ± s.d., and the error bars represent the means of three independent experiments or four independent biological mouse samples. Statistical significance was determined by a Student’s *t*-test. *P < 0.05, **P < 0.01 and ***P < 0.001. n.s., no significance.

To determine the role of miR-181a-5p in the fibrotic PHx model, we used AAV6 carrying the double-stranded CMV bGlobin-eGFP-U6-mmu-miR-181a-5p TuD (AAV6-miR-181a-5p inhibitor) to transiently block miR-181a-5p expression in HSCs in vivo, following previous studies that showed organ tropism for the activated HSCs of AAV6 (ref. ^5^). Because AAV6 was designed to simultaneously express GFP, we obtained fluorescence images and found that the AAV6 was specifically aggregated in the liver (Supplementary Fig. 4a). Statistical analysis of GFP^+^ cells to further assess the efficacy of AAV6 transduction in HSCs revealed that approximately 23% of the HSCs contained the AAV6-miR-181a-5p inhibitor (Supplementary Fig. 4b). HSCs were subsequently isolated to confirm that treatment with the AAV6-miR-181a-5p inhibitor significantly reduced the level of miR-181a-5p in the HSCs (Supplementary Fig. 4c). After three weeks of treatment with the AAV6-miR-181a-5p inhibitor, we used these mice to prepare a fibrotic PHx model, and a schematic diagram of the animal experiment is shown in Fig. 3a. Treatment with the AAV6-miR-181a-5p inhibitor suppressed the increases in ALT, AST and LDH levels and conversely increased the LW/BW ratio (Fig. 3b, c). The number of mitotic hepatocytes, Ki67 staining and PCNA expression were clearly greater in the AAV6-miR-181a-5p inhibitor-treated group than in the control group (Fig. 3d–h). In addition, we also isolated HSCs from each group to detect the expression of HGF. As expected, the RT‒qPCR and western blotting results revealed that the AAV6-miR-181a-5p inhibitor promoted the expression of HGF in HSCs (Fig. 3i, j). In summary, miR-181a-5p enrichment in activated HSCs suppressed liver regeneration in fibrotic mice via downregulation of HGF expression.

### Characterization of MSCs and MSC-EVs

To our knowledge, an effective therapeutic approach for promoting fibrotic liver regeneration that adapts to clinical translational applications is still lacking. MSC-EVs have been previously demonstrated to promote the regeneration of various organs. We recently revealed the role of MSC-EVs in improving the proliferative ability of senescent hepatocytes^13^. Therefore, we hypothesized that MSC-EVs may also have promising potential in promoting fibrotic liver regeneration.

**Fig. 4:**
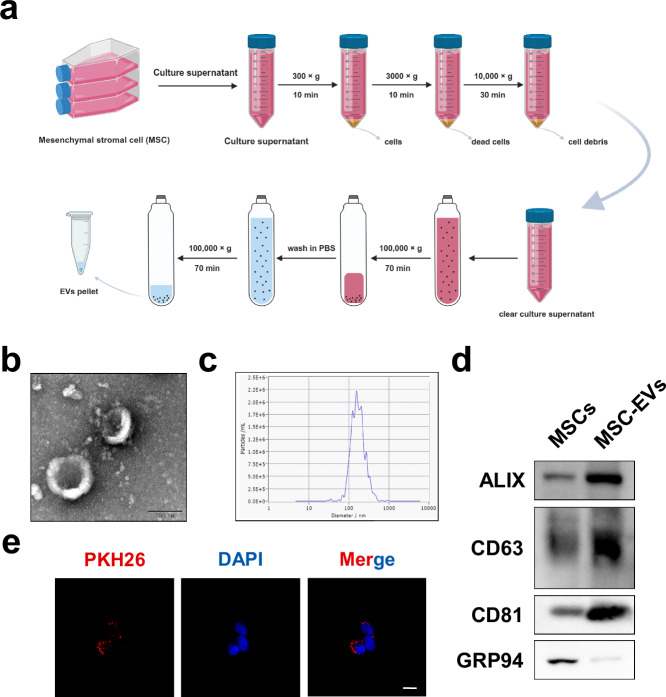
Characterization of MSC-EVs. **a** A schematic diagram of the procedures for obtaining MSC-EVs from MSC-conditioned medium. **b**, **c** TEM (b) and NTA (c) were used to detect the morphology and size of MSC-EVs. Scale bar, 0.1 μm. **d** Representative western blotting results showing the expression of specific vesicle-related markers, namely, ALIX, CD63, CD81 and GRP94. **e** The representative fluorescence images of the uptake of MSC-EVs (fluorescent red) in HSCs were obtained via confocal microscopy. Scale bar, 20 μm.

First, we isolated MSCs from umbilical cords, which were visualized as fibroblast-like cells (Supplementary Fig. 5a). Flow cytometry analysis revealed that the cells were negative for CD11b, CD19, CD34, CD45 and HLA-DR but positive for CD73, CD90 and CD105 (Supplementary Fig. 5b). We also observed that the obtained cells have the potential to differentiate into adipogenic and osteogenic cells (Supplementary Fig. 5a). After ultracentrifugation, the MSC-EVs were purified from MSC-conditioned medium (MSC-CM) (Fig. 4a), which featured round-shaped particles with a bilayer membrane, a size ranging from 30 to 150 nm, positive expression of exosomal markers (CD63, CD81 and ALIX) and negative expression of GRP94, verifying that the purity of our sample was not contaminated with ER-derived vesicles (Fig. 4b‒d). We subsequently labeled MSC-EVs with PKH26 followed by coculture with activated HSCs for 6 h and observed that the MSC-EVs (red dots) were located in the cytoplasm of the HSCs, indicating that the MSC-EVs could be taken up by activated HSCs (Fig. 4e).

### The biodistribution of MSC-EVs in the fibrotic PHx model

**Fig. 5:**
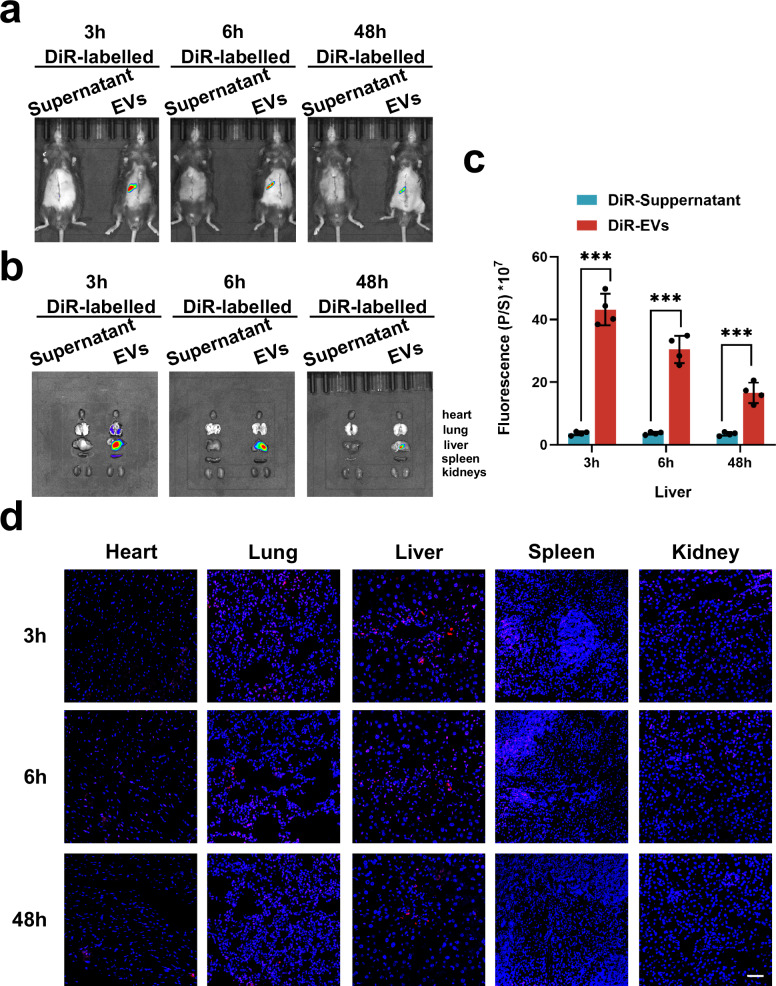
The biodistribution of MSC-EVs in fibrotic PHx model mice. **a** The representative in vivo imaging system (IVIS) images of the fibrotic PHx mouse model injected with DiR supernatant-treated control (left) or DiR-labeled MSC-EVs (right) at 3, 6 and 48 h. **b** Representative IVIS images of organs (from top to bottom: heart, lung, liver, spleen and kidneys) collected at 3, 6 and 48 h. **c** A quantification of the fluorescence intensity in livers at 3, 6 and 48 h (*n* = 4 independent biological mouse samples). **d** The representative fluorescent sections of different organs (heart, lung, liver, spleen and kidneys) used to investigate the biodistribution of DiR-labeled MSC-EVs (red). Scale bar, 50 μm. The statistical data are presented as mean ± s.d., and the error bars represent the means of four independent biological mouse samples. Statistical significance was determined by a Student’s *t*-test. *P < 0.05, **P < 0.01 and ***P < 0.001.

To investigate the biodistribution of MSC-EVs in the fibrotic PHx model, we labeled MSC-EVs with DiR dye and treated them in the fibrotic PHx model through the caudal vein. In vivo fluorescence images were taken at 3, 6 and 48 h after the MSC-EVs were transferred. The fluorescence signal was found to be located mainly in the residual liver at these three time points (Fig. 5a). After the mice were sacrificed, five pieces of fresh tissue, including heart, lung, liver, spleen and kidney, were collected to image, which revealed that most of the MSC-EVs were concentrated in the liver (Fig. 5b, c). These results further confirmed that the majority of DiR-labeled MSC-EVs were localized in the livers of the fibrotic PHx model mice (Fig. 5d).

### MSC-EVs promote fibrotic liver regeneration via the upregulation of HGF expression in HSCs

**Fig. 6:**
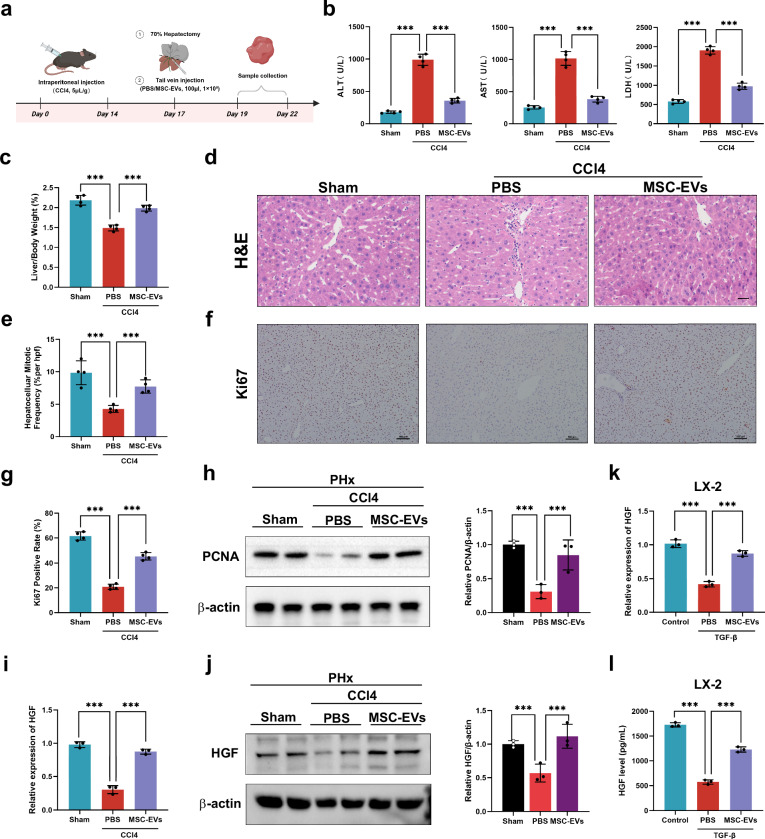
MSC-EVs promote fibrotic liver regeneration via the upregulation of HGF expression in HSCs. **a** A schematic diagram of the application of MSC-EVs to determine whether MSC-EVs can stimulate the regeneration of the fibrotic liver. **b** The serum ALT, AST and LDH levels in different treatment groups (*n* = 4 independent biological mouse samples). **c** The LW/BW ratio in different treatment groups (*n* = 4 independent biological mouse samples). **d** The representative images of H&E-stained samples from different treatment groups. Scale bar, 50 μm). **e** A quantification of the mitotic frequency determined by counting the numbers of mitotic nuclei and total nuclei in randomly selected fields (*n* = 4 independent biological mouse samples). **f**, **g** The representative images (**f**) and quantification (**g**) of Ki67 immunohistochemical staining in different treatment groups. Scale bar, 100 μm (*n* = 4 independent biological mouse samples). **h** Western blotting (left) and relative quantification (right) showing the expression of PCNA in different treatment groups (*n* = 3 independent experiments). **i**, **j** RT‒qPCR (**i**) and representative western blotting (**j**, left) results showing the expression of HGF in HSCs isolated from different treatment groups and a quantification of HGF protein levels (**j**, right) (*n* = 3 independent experiments). In vitro experiments including LX-2 HSCs treated with TGF-β were given PBS or MSC-EVs. **k**, **l** RT‒qPCR (**k**) and ELISA (**l**) were used to measure the HGF level in the LX-2 HSC line (*n* = 3 independent cell experiments). The statistical data are presented as mean ± s.d., and the error bars represent the means of three independent experiments or four independent biological mouse samples. Statistical significance was determined by a Student’s *t*-test. *P < 0.05, **P < 0.01 and ***P < 0.001.

Next, we evaluated the ability of MSC-EVs to promote fibrotic liver regeneration. We first prepared a mouse fibrotic PHx model (Fig. 6a) as described above and then, through the tail vein, injected the mice with MSC-EVs (100 µl, approximately 1 × 10^8^ nanoparticles) or PBS (100 µl). As shown in Fig. 6b, the results revealed the ability of MSC-EVs to restore liver function, as they significantly reduced the levels of serum hepatic enzymes (ALT, ALT and LDH) at 48 h after the preparation of the fibrotic PHx model. PSR staining and western blotting assays for detecting the expression of α-SMA and Vimentin were conducted to measure the severity of hepatic fibrosis. As expected, the collagen-stained areas were associated with the expression of α-SMA and Vimentin in the liver tissues, which was consistent with previous studies^14,15^. MSC-EV treatment significantly decreased the collagen-stained area and the expression of α-SMA and Vimentin compared with those in the PBS group (Supplementary Fig. 6a, b). In addition, we found that the decrease in the LW/BW ratio caused by fibrosis was markedly reversed after treatment with MSC-EVs (Fig. 6c). The results of H&E staining also revealed that treatment with MSC-EVs increased the number of mitotic hepatocytes in this model (Fig. 6d, e). Compared with the control, MSC-EVs increased the ratio of Ki67-positive cells and upregulated the expression of PCNA, suggesting that MSC-EVs dramatically strengthened the weakened potential for liver regeneration in the fibrotic PHx model mice (Fig. 6f–h).

We also isolated HSCs from the fibrotic PHx model mice in each group, and the RT‒qPCR and western blotting results revealed that MSC-EVs promoted the expression of HGF in HSCs compared with that in the control groups (Fig. 6i, j). In the in vitro study, TGF-β-treated HSCs presented low HGF expression and release, while MSC-EVs treatment reversed these effects (Fig. 6k, l). In summary, these data suggest that MSC-EVs promote fibrotic liver regeneration by increasing the synthesis and secretion of HGF by HSCs.

### LncEEF1G derived from MSC-EVs upregulates HGF expression in HSCs by acting as a sponge of miR-181a-5p

**Fig. 7:**
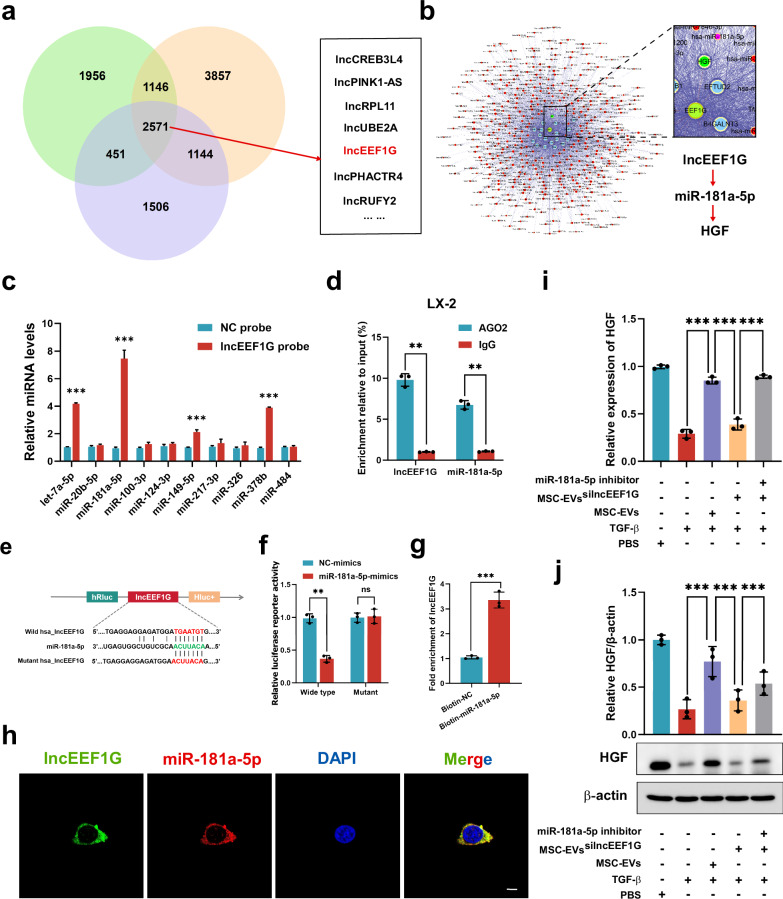
LncEEF1G derived from MSC-EVs upregulates HGF expression in HSCs by acting as a sponge to target miR-181a-5p. **a** A Venn diagram showing lncRNAs from three independent samples of MSC-EVs obtained through whole-transcriptome sequencing analysis. **b** Bioinformatic analysis revealing that lncEEF1G acts as a miRNA sponge for miR-181a-5p in HSCs. **c** RT‒qPCR analysis of the miRNAs (let-7a-5p, miR-20b-5p, miR-100-3p, miR-124-3p, miR-149-5p, miR-217-3p, miR-326, miR-378b, miR-484 and miR-181a-5p) that potentially interact with lncEEF1G (*n* = 3 independent cell experiments). **d** A RIP assay was conducted to confirm the interaction between lncEEF1G and miR-181a-5p (*n* = 3 independent cell experiments). **e** The RNAalifold online database was used to predict the site at which lncEEF1G binds to miR-181a-5p for further mutation. **f** The luciferase activity of WT and mutant lncEEF1G was detected by luciferase reporter assays after cotransfection with either miR-181a-5p mimics or control mimics (*n* = 3 independent cell experiments). **g** A biotin-labeled miR-181a-5p probe was used to confirm that lncEEF1G could be captured by miR-181a-5p (*n* = 3 independent cell experiments). **h** FISH results showing the localization of lncEEF1G (green) and miR-181a-5p (red) in HSCs. Scale bar, 20 μm. For HSCs, PBS, TGF-β, TGF-β + MSC-EVs, TGF-β + MSC-EVs^silncEEF1G^ or TGF-β + MSC-EVs^silncEEF1G^ + miR-181a-5p were added to each group. This finding further verified that lncEEF1G in MSC-EVs regulates HGF levels by interacting with miR-181a-5p. **i**, **j** RT‒qPCR (**i**) and representative western blotting (**j**, upper) results showing the expression of HGF in HSCs in different treatment groups and a quantification (**j**, down) of HGF protein levels (*n* = 3 independent cell experiments). The statistical data are presented as mean ± s.d., and the error bars represent the means of three independent experiments. Statistical significance was determined by a Student’s *t*-test. *P < 0.05, **P < 0.01 and ***P < 0.001. n.s., no significance.

MSC-EVs contain multiple functional components, which could serve as important carriers for cell‒cell communication. We previously reported that MSC-EVs not only attenuate HIRI through their inner functional mitochondria, CCT2 and MnSOD but also improve the proliferative potential of senescent hepatocytes via enrichment of DDX5 (refs. ^10–13^). Therefore, in this study, we perform whole-transcriptome sequencing of MSC-EVs on the basis of the observation that miR-181a-5p in activated HSCs negatively affects fibrotic liver regeneration. With three biological replicates, we identified 2,571 lncRNAs enriched in MSC-EVs. Among them, lncEEF1G attracted our attention because bioinformatic analysis predicted that it could act as a sponge to directly bind to miR-181a-5p through (Fig. 7a, b). In addition, an online database (RegRNA 2.0, http://regrna2.mbc.nctu.edu.tw/) also predicted nine other putative miRNAs that could interact with lncEEF1G, namely, let-7a-5p, miR-20b-5p, miR-100-3p, miR-124-3p, miR-149-5p, miR-217-3p, miR-326, miR-378b and miR-484. An RNA pulldown assay was conducted to evaluate whether lncEEF1G could directly bind to these candidate miRNAs, which revealed that miR-181a-5p was the most highly enriched miRNA interacting with lncEEF1G in HSCs (Fig. 7c). As miRNAs can serve as RNA-induced silencing complex components to interact with Argonaute-2 (AGO2), we performed RNA immunoprecipitation (RIP) assays with an anti-AGO2 antibody and found that both miR-181a-5p and lncEEF1G could be pulled down by AGO2 (Fig. 7d). Moreover, we also predicted the site of lncEEF1G binding to miR-181a-5p via another online database (RNAalifold, http://rna.tbi.univie.ac.at/) and the lncEEF1G WT luciferase reporter vector, as well as the lncEEF1G mutant luciferase reporter vector encompassing the sequence that could not interact with miR-181a-5p, were constructed (Fig. 7e). In addition, RNAalifold was used to predict the secondary structure of lncEEF1G (Supplementary Fig. 7a). On the basis of the above constructed plasmids, dual-luciferase reporter assays were performed, and the results (Fig. 7f) revealed that the miR-181a-5p mimics noticeably suppressed the activity of the lncEEF1G WT luciferase reporter but did not affect the lncEEF1G mutant luciferase reporter. Moreover, a biotin-labeled miR-181a-5p probe was used to confirm that lncEEF1G could be captured by miR-181a-5p (Fig. 7g). FISH assays also demonstrated their colocalization in the cytoplasm of MSCs (Fig. 7h).

Next, a lentivirus carrying lncEEF1G-siRNA was used to downregulate lncEEF1G expression in MSCs, namely, MSCs^silncEEF1G^ (Supplementary Fig. 8a). The results indicated that silencing lncEEF1G in MSCs did not affect viability or MSC-related characteristics (Supplementary Fig. 8b–d). We subsequently performed a series of ultracentrifuge steps to isolate MSC-EVs^silncEEF1G^, which presented low lncEEF1G expression and retained EV-related features (Supplementary Fig. 8e–g). Then, we cocultured these MSC-EVs with HSCs under the indicated treatments and found that, compared with that of the MSC-EVs group, the ability of the MSC-EVs^silncEEF1G^ to promote the expression and secretion of HGF in activated HSCs was weak, whereas the combined use of miR-181a-5p inhibitors reversed this effect (Fig. 7i, j). In summary, these data suggest that lncEEF1G, which is highly expressed in MSC-EVs, can act as a sponge of miR-181a-5p to inhibit the combination of miR-181a-5p and HGF, eventually increasing the expression of HGF in HSCs.

### LncEEF1G derived from MSC-EVs strengthens liver regeneration in fibrotic PHx model mice

**Fig. 8:**
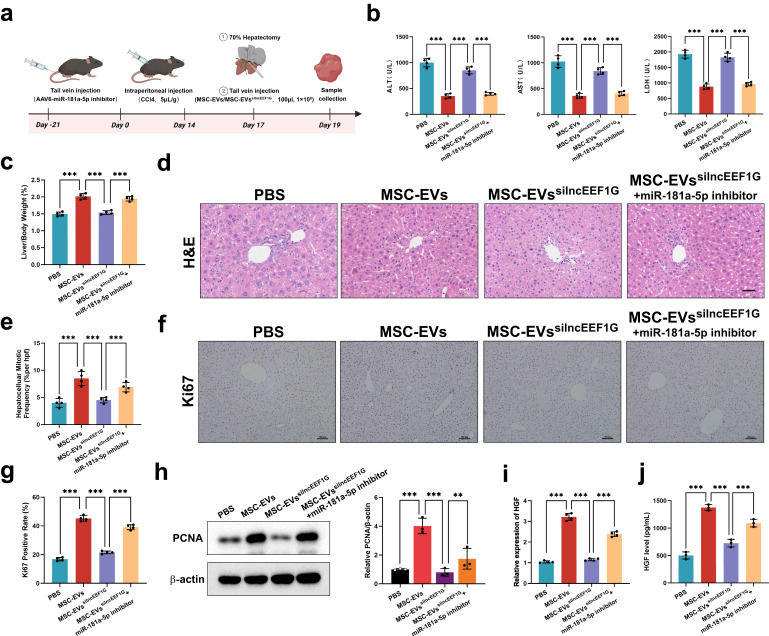
LncEEF1G derived from MSC-EVs strengthens liver regeneration in fibrotic PHx model mice. **a** A schematic diagram of the application of MSC-EVs^silncEEF1G^ to determine whether the ability of MSC-EVs to stimulate fibrotic liver regeneration depends on the direct interaction of lncEEF1G with miR-181a-5p to modulate HGF expression in HSCs. **b** Serum ALT, AST and LDH levels in different treatment groups (*n* = 4 independent biological mouse samples). **c** The LW/BW ratio in different treatment groups (*n* = 4 independent biological mouse samples). **d** The representative images of H&E-stained samples from different treatment groups. Scale bar, 50 μm). **e** A quantification of the mitotic frequency determined by counting the numbers of mitotic nuclei and total nuclei in randomly selected fields (*n* = 4 independent biological mouse samples). **f**, **g** The representative images (**f**) and quantification (**g**) of Ki67 immunohistochemical staining in different treatment groups. Scale bar, 100 μm (*n* = 4 independent biological mouse samples). **h** Western blotting (left) and relative quantification (right) showing the expression of PCNA in different treatment groups (*n* = 3 independent experiments). **i**, **j** RT‒qPCR (**i**) and ELISA (**j**) results showing the HGF levels of primary HSCs from each group (RT‒qPCR, *n* = 4 independent cell experiments; ELISA, *n* = 3 independent cell experiments). The statistical data are presented as mean ± s.d., and the error bars represent the means of three independent experiments or four independent biological mouse samples. Statistical significance was determined by a Student’s *t*-test. *P < 0.05, **P < 0.01 and ***P < 0.001.

Finally, we applied MSC-EVs^silncEEF1G^ to determine whether the ability of MSC-EVs to stimulate fibrotic liver regeneration depended on the interaction of lncEEF1G with miR-181a-5p to modulate HGF expression in HSCs. We used an AAV6-miR-181a-5p inhibitor to specifically decrease the level of miR-181a-5p in HSCs, followed by the preparation of a fibrotic PHx model and subsequent transfusion of MSC-EVs^silncEEF1G^ through the tail vein (Fig. 8a). As shown in Fig. 8b, c, knockdown of lncEEF1G partially weakened the hepatoprotective role of MSC-EVs and noticeably impaired their potential to increase the LW/BW ratio, whereas combination with the AAV6-miR-181a-5p inhibitor reversed these phenomena. In addition, treatment with MSC-EVs^silncEEF1G^ significantly suppressed the fibrotic liver regeneration promoted by MSC-EVs, as evidenced by a decrease in hepatocellular mitotic activity, Ki67 staining and PCNA expression, which were reversed by the addition of the AAV6-miR-181a-5p inhibitor (Fig. 8d–h). The results of RT‒qPCR and ELISAs with isolated HSCs subjected to the indicated treatments also revealed that the expression of HGF in the MSC-EVs^silncEEF1G^ group was lower than that in the MSC-EVs group and was inversely increased in the MSC-EVs^silncEEF1G^ + AAV6-miR-181a-5p inhibitor group (Fig. 8i, j).

### Engineered MSC-EVs with high lncEEF1G expression further upregulate HGF expression in HSCs to stimulate fibrotic liver regeneration

**Fig. 9:**
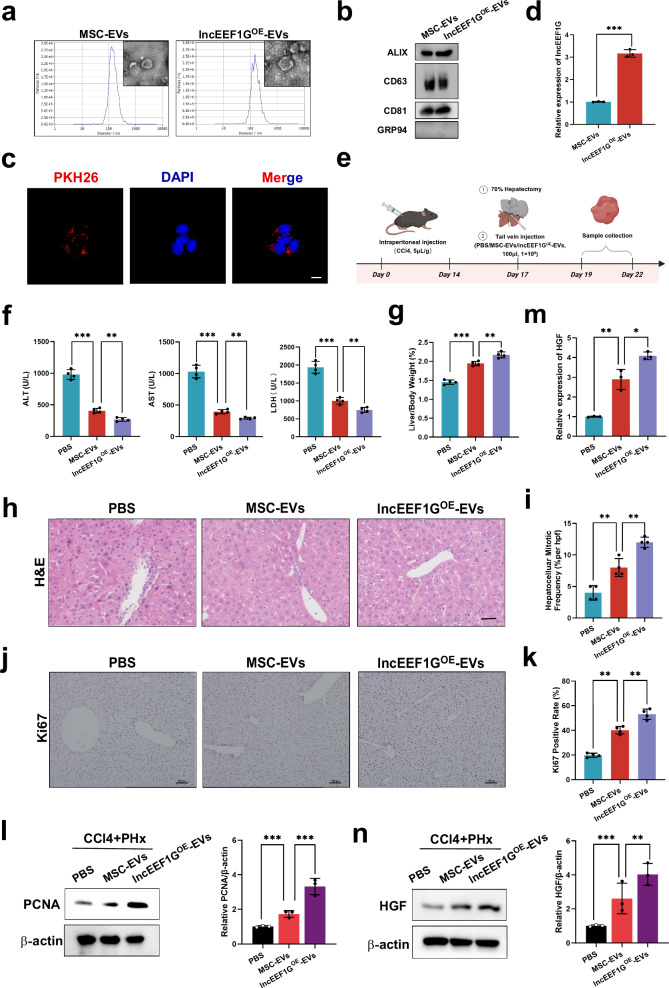
Engineered MSC-EVs with high lncEEF1G expression further upregulate HGF expression in HSCs to stimulate fibrotic liver regeneration. **a** TEM and NTA were used to detect the morphology and size of the MSC-EVs and lncEEF1G^OE^-EVs. Scale bar, 0.1 μm. **b** Representative western blots of specific vesicle-related markers (ALIX, CD63, CD81 and GRP94). **c** Representative fluorescence images of the uptake of lncEEF1G^OE^-EVs (fluorescent red) in HSCs were obtained via confocal microscopy. Scale bar, 20 μm). **d** RT‒qPCR showing the expression of lncEEF1G in MSC-EVs and lncEEF1G^OE^-EVs. **e** A schematic diagram of the application of lncEEF1G^OE^-EVs to detect whether lncEEF1G^OE^-EVs stimulate fibrotic liver regeneration. **f** Serum ALT, AST and LDH levels in different treatment groups (*n* = 4 independent biological mouse samples). **g** The LW/BW ratio in different treatment groups (*n* = 4 independent biological mouse samples). **h** The representative images of H&E-stained samples from different treatment groups. Scale bar, 50 μm). **i** A quantification of the mitotic frequency determined by counting the numbers of mitotic nuclei and total nuclei in randomly selected fields (*n* = 4 independent biological mouse samples). **j**, **k** The representative images (**j**) and quantification (**k**) of Ki67 immunohistochemical staining in different treatment groups. Scale bar, 100 μm (*n* = 4 independent biological mouse samples). **l** Western blotting (left) and relative quantification (right) showing the expression of PCNA in liver samples subjected to different treatments (*n* = 3 independent experiments). **m**, **n** RT‒qPCR (**m**) and representative western blotting (**n**, left) results showing the expression of HGF in HSCs isolated from different treatment groups and a quantification (**n**, right) of HGF protein levels (*n* = 3 independent experiments). The statistical data are presented as mean ± s.d., and the error bars represent the means of three independent experiments or four independent biological mouse samples. Statistical significance was determined by a Student’s *t*-test. *P < 0.05, **P < 0.01 and ***P < 0.001.

Based on the effects of lncEEF1G discussed above, we used a lncEEF1G-overexpressing lentiviral vector to construct engineered MSC-EVs with high expression of lncEEF1G (lncEEF1G^OE^-EVs) to increase the ability of MSC-EVs to promote fibrotic liver regeneration. Compared with the control, the lncEEF1G-overexpressing lentiviral vector did not affect the surface features of the MSCs or their ability to differentiate into multiple lineages but dramatically upregulated lncEEF1G expression in the MSCs (Supplementary Fig. 9a–d). Then, lncEEF1G^OE^-EVs were purified from lncEEF1G^OE^-MSC-CM. No significant differences in morphology, number of nanoparticles or EV-specific markers were observed between MSC-EVs (control-EVs) and lncEEF1G^OE^-EVs (Fig. 9a, b). HSCs also took up lncEEF1G^OE^-EVs (Fig. 9c). In addition, RT‒qPCR further confirmed that the content of lncEEF1G was greater than that in the control-EVs (Fig. 9d).

Next, we compared the therapeutic efficacy of lncEEF1G^OE^-EVs with that of MSC-EVs in promoting fibrotic liver regeneration (Fig. 9e). As shown in Fig. 9f, the levels of serum hepatic enzymes in the lncEEF1G^OE^-EVs group were markedly lower than those in the MSC-EVs group. In addition, we also found that lncEEF1G^OE^-EVs had a greater effect on stimulating fibrotic liver regeneration, as evidenced by the increased LW/BW ratio, the increased activity of hepatocellular mitosis, and the increased expression of Ki67 and PCNA (Fig. 9g-l). HSCs from each group were isolated for RT‒qPCR and Western blotting assays, which revealed that, compared with MSC-EVs treatment, lncEEF1G^OE^-EVs treatment further promoted the synthesis of HGF in HSCs (Fig. 9m, n). In summary, these results support the potential of lncEEF1GOE-EVs to increase HGF expression in HSCs to strengthen fibrotic liver regeneration.

## Discussion

Fibrosis leads to a reduced potential for liver regeneration, increasing the risk of small for size syndrome in patients receiving PH. However, due to the ambiguous underlying mechanism, there is still no effective treatment adapted for clinical translational application to promote fibrotic liver regeneration. In this study, we demonstrate that MSC-EVs can increase the synthesis and secretion of HGF in activated HSCs to increase liver regeneration in a fibrotic PHx model. Mechanistically, on the basis of the finding that miR-181a-5p, which is highly expressed in activated HSCs, plays a negative role in regulating HGF expression, high-throughput sequencing was performed and identified lncEEF1G, which is enriched in MSC-EVs and can be transferred into activated HSCs to comprehensively interact with miR-181a-5p to relieve its suppression of the target gene HGF. Furthermore, engineered lncEEF1G^OE^-EVs were constructed to further strengthen liver regeneration in fibrotic PHx model mice.

Hepatic fibrosis is a common and dynamic pathological process in the progression of various chronic liver diseases and is characterized by excess ECM production and fibrous connective tissue deposition. HSCs are the most concentrated subcluster and can secrete ECM and proinflammatory cytokines to induce fibrogenesis and cellular injury^20^. In addition, HSCs affect liver regeneration via the release of HGF and TGF-β^2^. Secretion of HGF is predominant in quiescent HSCs and promotes liver regeneration, whereas low expression of HGF in the activated form limits hepatocellular proliferation. In this study, we consistently validate this phenomenon in a fibrotic PHx mouse model. Therefore, exploring the underlying mechanism of low HGF expression in activated HSCs to formulate a therapeutic strategy is key for effectively preventing severe postoperative complications after PH.

Several factors, including miRNAs, which are small noncoding RNAs with lengths of 22–25 nt, have been demonstrated to regulate the function and status of HSCs. For example, miR-185, miR-30a, miR-122, miR-98 and miR-455-3p have been shown to suppress HSCs activation^21–25^, whereas miR-214, miR-130b-5p, miR-15b and miR-16 promote HSCs activation^26–28^. Based on these findings, we further detected the differential miRNAs between HSCs and activated HSCs via miRNA microarray profile analysis and identified miR-181a-5p among the highly expressed miRNAs in activated HSCs because the target sequence of miR-181a-5p within the HGF-3′UTR is responsible for maintaining the stability of HGF mRNA. miR-181a-5p is a widely analyzed miRNA that has been shown to have bidirectional potential in the progression of various types of cancer (negative for hepatocellular carcinoma, lung cancer, colorectal cancer and melanoma^29–32^ and positive for breast cancer, lymphoblastic leukemia and gastric cancer)^33–35^, as well as regulating the differentiation and viability of MSCs^36^, affecting acute cellular rejection after heart transplantation^37^ and aggravating HIRI^38^. Wang et al. reported that miR-181a-5p downregulates collagen production in HSCs by affecting TLR4/NF-κB signaling^39^. In this study, we find that miR-181a-5p could also suppress the expression and secretion of HGF in HSCs by binding to the 3′UTR of HGF mRNA. Moreover, an AAV6-miR-181a-5p inhibitor that specifically downregulated miR-181a-5p in HSCs was used for an in vivo study to further verify that blocking miR-181a-5p strengthened the potential of HSCs to secrete HGF to promote liver regeneration in the fibrotic PHx model mice, indicating that miR-181a-5p may be a therapeutic target for fibrotic liver regeneration.

Due to their various immunomodulatory abilities (for example, organ injury repair and tissue regeneration), MSCs have been identified as promising treatments for liver diseases. EVs are increasingly being studied because they constitute a primary approach by which MSCs perform their biological functions. Owing to their small volume and similar characteristics to those of parental MSCs, MSC-EVs are more suitable for clinical application because of their convenient storage advantages and low incidence of tumorigenicity and pulmonary embolism. Our group previously reported the hepatoprotective potential of MSC-EVs in HIRI and recently revealed their role in attenuating senescence-related damage as well as enhancing liver regeneration in an aged PHx mouse model^13^. In addition, we found that MSC-EVs promoted fibrotic liver regeneration, and we further focused on their effect on HSCs, which revealed that MSC-EVs not only attenuated the activation of HSCs, which is consistent with the findings of previous studies, but also promoted the synthesis and secretion of HGF by HSCs.

Recently, an increasing number of studies have revealed the potential of lncRNAs in regulating the activation of HSCs. Liu et al. reported that cholangiocyte-derived exosomes from chronic cholestatic liver diseases stimulate the activation of HSCs by transferring lncRNA-H19 (ref. ^40^). Zhan et al. reported that lncRNA-MIAT regulates the Hippo pathway to activate HSCs^13^. Yu et al. demonstrated that lncRNA-SNHG7 promotes HSCs activation by regulating the miR-278a-3p/DVL2 pathway^25^. In contrast, other studies have validated the role of lncRNA-p21 and lncRNA GAS5 in restraining the progression of hepatic fibrosis^41,42^. For further mechanistic exploration, high-throughput sequencing was performed on the basis of the observation that MSC-EVs have biological potential, primarily because of their functional contents. We found many lncRNAs enriched in MSC-EVs and, among these, focused on lncEEF1G as it was predicted to act as a competing endogenous RNA to bind miR-181a-5p and inhibit the combination of miR-181a-5p and HGF mRNAs, constituting a lncRNA‒miRNA‒mRNA regulatory network. LncEEF1G, a 2.4 kb lncRNA, is a novel lncRNA transcribed from the eukaryotic translation elongation factor 1 gamma (*EEF1G*) gene, which is located at chromosome 11q12.3 and can be translated into a family of the EF1 complex to regulate protein synthesis in the elongation phase^43^. The results of the luciferase reporter assay, RIP, FISH and biotin-labeled miRNA capture, which were consistent with the prediction, confirmed the role of lncEEF1G in binding to miR-181a-5p to suppress its potential. In addition, MSC-EVs^silncEEF1G^ combined with the AAV6-miR-181a-5p inhibitor were used for compensatory experiments, which revealed that the knockdown of lncEEF1G impaired the potential of MSC-EVs to increase the synthesis and release of HGF in HSCs and promote fibrotic liver regeneration, whereas the combined inhibition of miR-181a-5p blocked these phenomena.

Finally, based on the therapeutic effect of lncEEF1G on fibrotic liver regeneration, we utilized a gene modification approach to produce lncEEF1G^OE^-EVs, as lentivirus transduction is a convenient, efficient and safe method, and MSC-EVs have been demonstrated to be ideal vehicles that have biological safety, good biocompatibility and high efficiency of cell uptake^44,45^. In the past, Fang et al. demonstrated the ability of engineered MSC-EVs with high SHP2 expression to promote mitophagy to treat Alzheimer’s disease^46^. Han et al. reported that overexpressing miR-214-3p in MSC-EVs promoted myocardial repair^47^. In this study, after confirming that lentivirus transduction did not affect viability or stem-cell-related features, lncEEF1G^OE^-EVs were purified to treat fibrotic PHx model mice, which revealed that they were safely transfused into animals and were superior to control-EVs in upregulating HGF expression in HSCs and promoting fibrotic liver regeneration.

In summary, the present study reveals a novel effect of MSC-EVs in promoting fibrotic liver regeneration. Mechanistically, this effect may be partially attributed to the enrichment of lncEEF1G in MSC-EVs, which acts as a competing endogenous RNA for miR-181a-5p to affect its activation and subsequently promote the synthesis and secretion of HGF in HSCs. These results also suggest that the use of MSC-EVs may become an attractive therapeutic approach for reducing postoperative complications and mortality after patients experience PH.

## Supplementary information

Supplementary Information
