# Supplementary material for: Engineered EVs from LncEEF1G - overexpressing MSCs promote fibrotic liver regeneration by upregulating HGF release from hepatic stellate cells
Source: Exp Mol Med. 2025 Mar 3;57(3):584–600. doi: 10.1038/s12276-025-01413-4 (PMC11958733; doi:10.1038/s12276-025-01413-4)
Supplement: Supplementary file 1 — Supplementary Information [file 12276_2025_1413_MOESM1_ESM.pdf]

Supporting information

Engineered Extracellular Vesicles from LncEEF1G-Overexpressing Mesenchymal Stem Cells to Promote Fibrotic Liver Regeneration by Upregulating HGF Release from Hepatic Stellate Cells

Jiebin Zhang[1, 2, *], Xiaotong Qiu[1, 2, *], Yunguo Lei[1, 2, 6, *], Haitian Chen[1, 2, *], Dongwei Wu[1, 2,] Tingting Wang[1,2,] Xin Sui[5], Jiaqi Xiao[1, 2], Chenhao Jiang[1, 2], Huayao Zhang[8], Yasong Liu[1, 2], Xiaoquan Liu[4], Yingcai Zhang[1, 2], Xu Che[7], Ye Lin[3], Jia Yao[1, 2#], Zihao Pan[3, #], Rong Li[2 ,#], Jun Zheng[1, 2, #]

Table of Contents

Fig. S1…………………………………………………………………………….3

Fig. S2…………………………………………………………………………….4

Fig. S3…………………………………………………………………………….5

Fig. S4…………………………………………………………………………….6

Fig. S5…………………………………………………………………………….7

Fig. S6…………………………………………………………………………….8

Fig. S7…………………………………………………………………………….9

Fig. S8……………………………………………………………………………10

Fig.S9…………………………………………………………………………… 12

Supplementary Table…………………………………………………………......14

Supplementary Figure 1. Extraction and identification of mouse primary hepatic stellate cells.

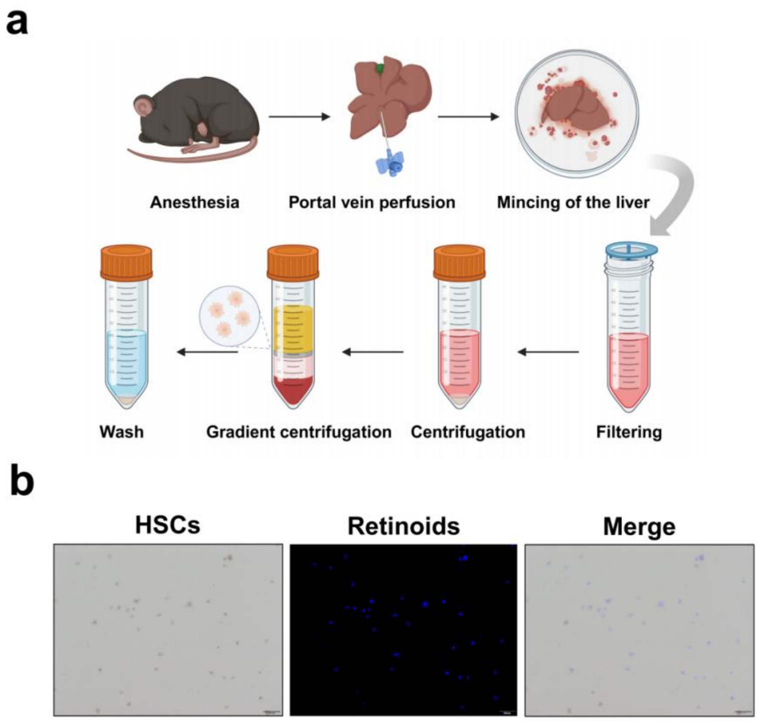

(a)Schematic diagram of extracting and obtaining primary hepatic stellate cells (HSCs) from mouse liver tissue. (b) Representative images of freshly isolated HSCs visualized using phase-contrast microscopy (left) and retinoid fluorescence (middle). A merged image (right) of the retinoid fluorescence with the phase-contrast image

Supplementary Figure 2. Establishment of a mouse liver fibrosis model via carbon tetrachloride (CCl4) treatment.

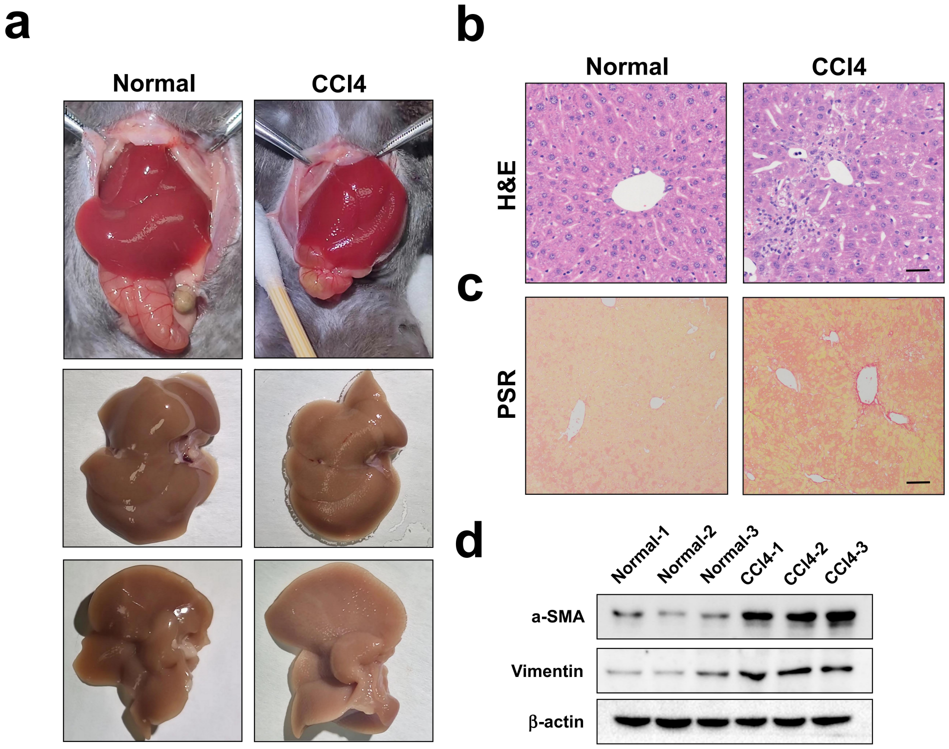

(a) Liver morphology of mice in the normal and CCl4 treatment group. (b) Representative images for H&E staining for the normal and CCl4 treatment group (Scale bar = 100 μm, n = 4 independent biological mice samples). (c) Representative liver sections for picrosirius red (PSR) staining of the two groups (Scale bar = 100 μm, n = 4 independent biological mice samples). (d) Western blotting assays showing the expression of α-SMA and vimentin in liver samples of each group.

Supplementary Figure 3. The expression of miR-181a-5p of hepatic stellate cells.

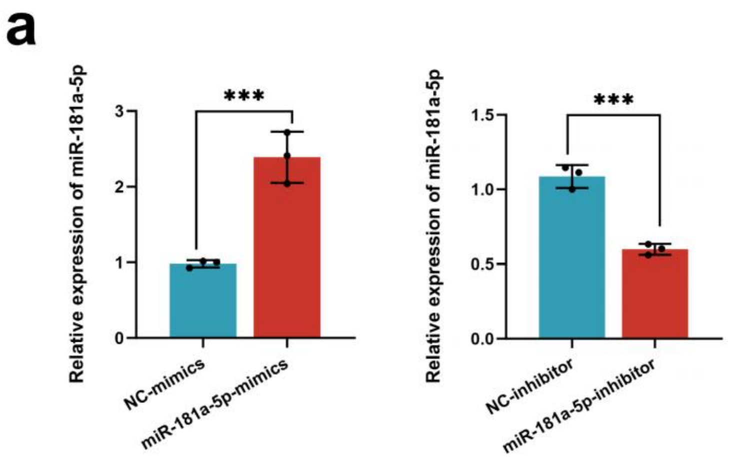

(a) The expression of miR-181a-5p in HSCs was upregulated or downregulated after being transfected with miR-181a-5p mimic or inhibitor, respectively. (n=3 independent cell experiments). Statistic data are presented as the mean ± SD, error bars represent the means of three independent experiments. Statistical significance was determined by student's t test. *p<0.05, **p<0.01, and ***p<0.001.

Supplementary Figure 4. Blocking miR-181a-5p expression of HSCs in vivo using AAV6.

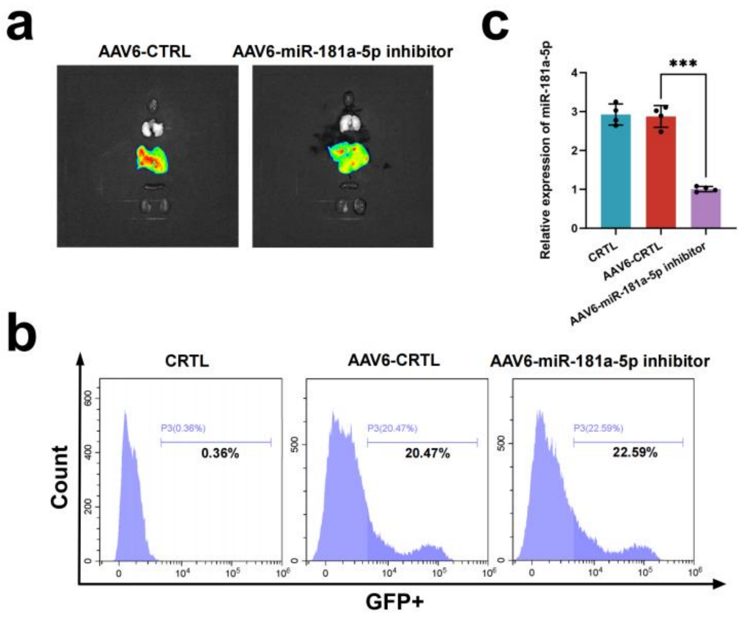

(a)Representative in vivo imaging system (IVIS) images of multiple organs in mice transfected with adeno-associated virus serotype 6 (AAV6) carrying double-stranded CMV bGlobin-eGFP-U6-mmu-miR-181a-5p TuD (AAV6-miR-181a-5p inhibitor) or AAV6-Control. (b) Flow cytometry analysis was performed to investigate the proportion of GFP-positive hepatic stellate cells (HSCs) after AAV6 transfection. (n=4 independent cell experiments). (c) RT-qPCR showing the miR-181a-5p levels of primary HSCs from AAV6 transfected mouse liver (n = 4 independent experiments). Statistical significance was determined by student's t test. *p<0.05, **p<0.01, and ***p<0.001. ns no significance.

Supplementary Figure 5. The biological features of MSCs.

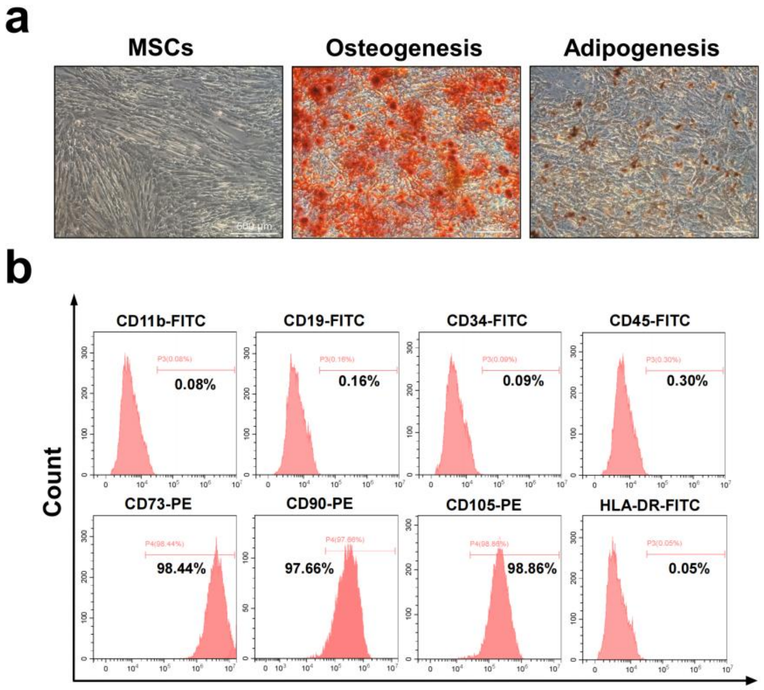

(a) The fibroblast-like morphology of umbilical cord-derived mesenchymal stem cells was observed under a light microscope (left; Scale bar = 500 μm). Representative images of MSCs differentiating into osteogenesis (middle) and adipogenesis (right) (Scale bar = 200 μm). (b) Flow cytometry analysis was performed to investigate the expressions of specific markers of MSCs, including CD11b, CD19, CD34, CD45, CD73, CD90, CD105, and HLA-DR.

Supplementary Figure 6. MSC-EVs alleviates liver fibrosis in the fibrotic PHx model.

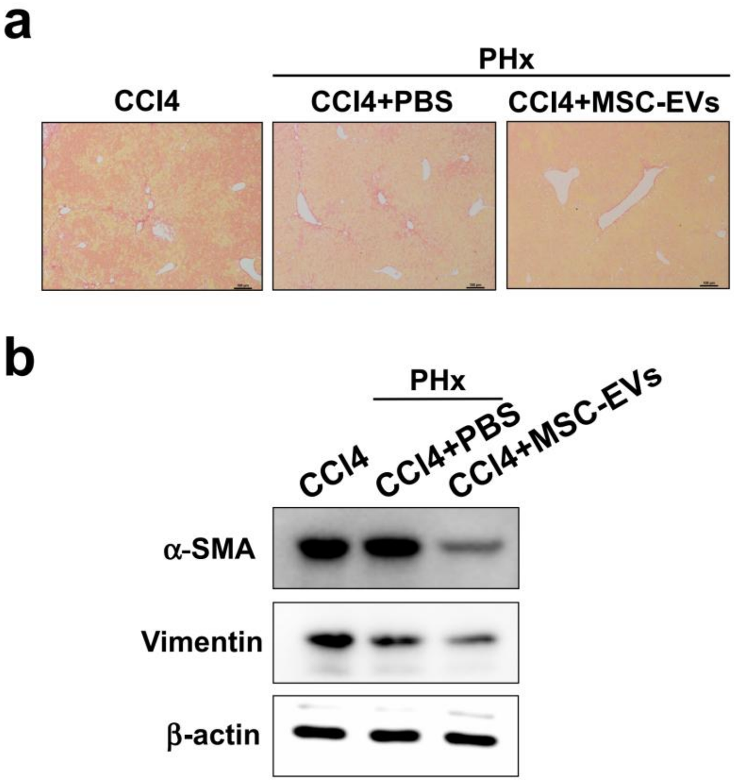

(a)Representative liver sections for picrosirius red (PSR) staining of each group (Scale bar = 100 μm, n = 4 independent biological mice samples). (b) Western blotting assays showing the expression of α-SMA and vimentin in liver samples of each group (n = 4 independent biological mice samples).

Supplementary Figure 7. The secondary structure of lncEEF1G.

a

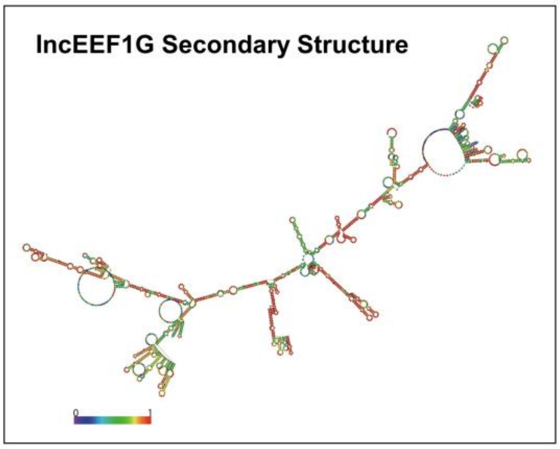

(a) The RNAalifold online tool was employed to predict the secondary structure of lncEEF1G.

Supplementary Figure 8. The expression of lncEEF1G in MSCs and MSC-EVs and the characterization of $MSCs^{silncEEF1G}$ and $MSC\text{-}EVs^{silncEEF1G}$.

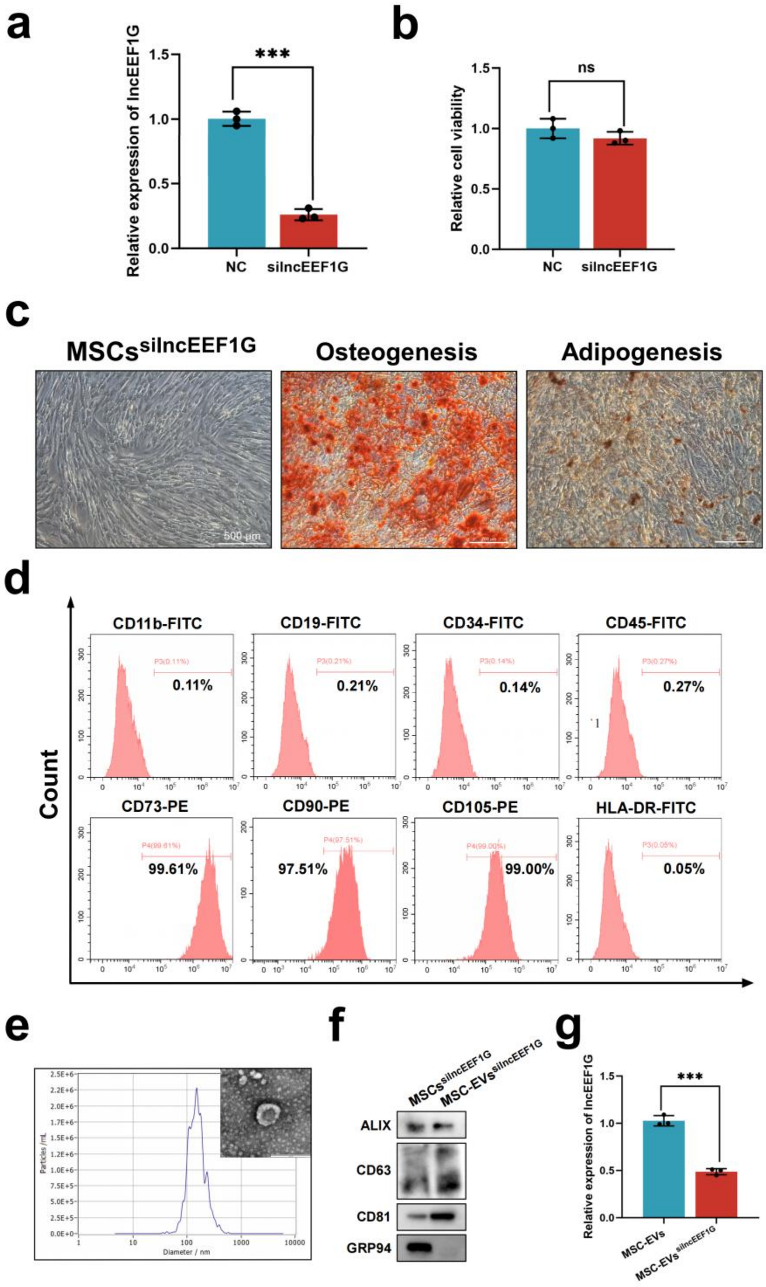

(a) RT-qPCR showing the lncEEF1G levels of MSCs in different treatment groups. (n=3 independent cell experiments). (b) Cell viability of MSCs in different treatment groups. (n=3 independent cell experiments). (c) The fibroblast-like morphology of

MSCs^{silncEEF1G} was observed under a light microscope (left, Scale bar = 500 μm). Representative images of MSCs^{silncEEF1G} differentiating into osteogenesis (middle) and adipogenesis (right) (Scale bar = 200 μm). (d) Flow cytometry analysis was performed to investigate the expressions of specific markers of MSCs^{silncEEF1G}, including CD11b, CD19, CD34, CD45, CD73, CD90, CD105, and HLA-DR. (e) Transmission electron microscopy (TEM) and nanoparticle tracking analysis (NTA) were used to detect the morphology and size of MSC-EVs^{silncEEF1G} (Scale bar = 0.1 μm). (f) Western blotting assays showing the expression of the specific vesicle-related markers (ALIX, CD63, CD81, and GRP94). (g) RT-qPCR showing the lncEEF1G levels in EVs of MSCs under different treatments. (n=3 independent cell experiments). Statistical significance was determined by students' t test. *$p<0.05$, **$p<0.01$, and ***$p<0.001$.

Supplementary Figure 9. The biological features of engineered MSCs with overexpressed lncEEF1G.

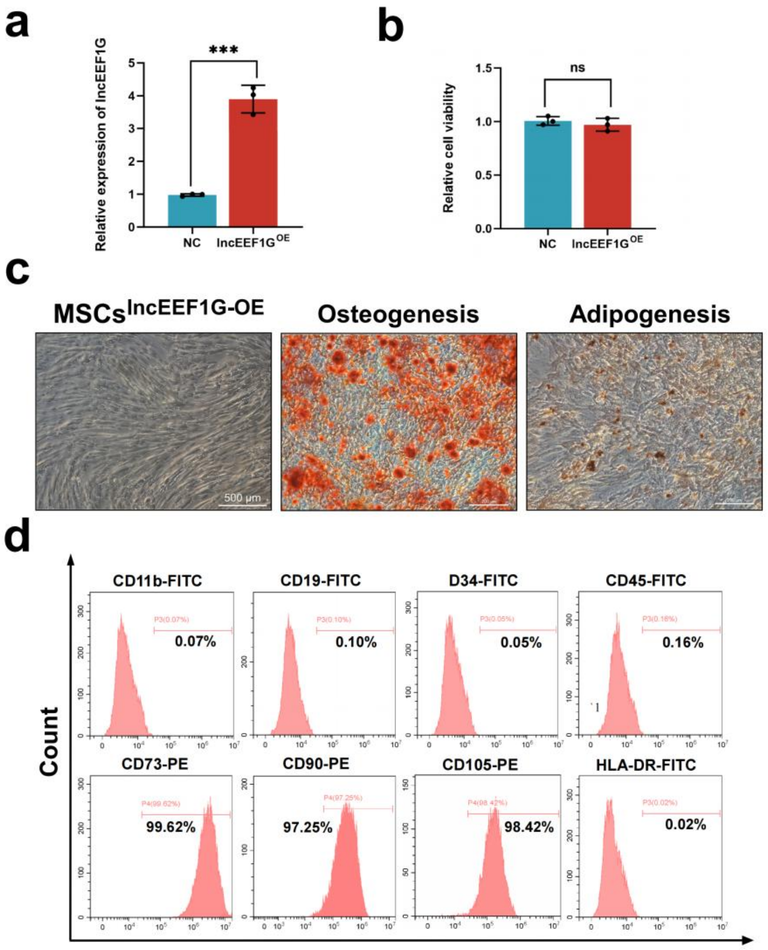

(a) RT-qPCR showing the lncEEF1G levels of MSCs with indicated treatments. (n=3 independent cell experiments). (b) Cell viability of MSCs in each groups. (n=3 independent cell experiments). (c) The fibroblast-like morphology of MSCs$^{lncEEF1G-OE}$ was observed under a light microscope (left, Scale bar = 500 μm). Representative images of MSCs$^{lncEEF1G-OE}$ differentiating into osteogenesis (middle) and adipogenesis (right) (Scale bar = 200 μm). (d) Flow cytometry analysis was performed to investigate the expressions of specific markers of MSCs$^{lncEEF1G-OE}$, including CD11b,

CD19, CD34, CD45, CD73, CD90, CD105, and HLA-DR. Statistical significance was determined by student's t test. *p<0.05, **p<0.01, and ***p<0.001. ns no significance.

Supplementary Table 1. Primer sequences for quantitative RT-PCR

Gene Symbol	Sequence direction	Sequence
Mouse-HGF	Forward primer	5’- ATGTGGGGGACCAAACTTCTG -3’
	Reverse primer	5’- GGATGGCGACATGAAGCAG -3’
Mouse- TGF-β	Forward primer	5’- CTCCCGTGGCTTCTAGTGC -3’
	Reverse primer	5’- GCCTTAGTTTGGACAGGATCTG -3’
Mouse-β-actin	Forward primer	5’- GGCTGTATTCCCCTCCATCG -3’
	Reverse primer	5’- CCAGTTGGTAACAATGCCATGT -3’
Human- HGF	Forward primer	5’- GAGAGTTGGGTTCTTACTGCACG -3’
	Reverse primer	5’- CTCATCTCCTCTTCCGTGGACA -3’
Human-GAPDH	Forward primer	5’- GTCTCCTCTGACTTCAACAGCG -3’
	Reverse primer	5’- ACCACCCTGTTGCTGTAGCCAA -3’
